# Supplementary material for: Clinical and economic burden of low back pain in low- and middle-income countries: a systematic review
Source: BMJ Open. 2023 Apr 25;13(4):e064119. doi: 10.1136/bmjopen-2022-064119 (PMC10151982; doi:10.1136/bmjopen-2022-064119)
Supplement: Supplementary data [file bmjopen-2022-064119supp001.pdf]

Appendix 1: Clinical and economic burden search strategy

Appendix 1a: Medline, CINAHL, Embase and AMED (inception to 10th December/2021

Search ID	Search Terms
S23	S21 AND S22
S22	S1 OR S2 OR S3 OR S4 OR S5 OR S6 OR S7 OR S8 OR S9 OR S10 OR S11 OR S12 OR S13 OR S14 OR S15 OR S16 OR S17 OR S18 OR S19 OR S20
S21	AB low back pain
S20	AB hospitalization
S19	AB cost of illness
S18	AB absenteeism
S17	AB ambulatory care
S16	AB drug cost
S15	AB emergency medical services
S14	AB healthcare services
S13	AB nursing services
S12	AB economics
S11	AB physician
S10	AB burden
S9	AB clinical impact
S8	AB utilization
S7	AB burden of illness
S6	AB cost
S5	AB nursing costs
S4	AB physician cost
S3	AB physician visits
S2	AB emergency department visits
S1	AB years lived with disability

Appendix 1b: Scopus (inception to 10th December/2021

Search ID	Search Terms
1	TITLE-ABS-KEY (low AND back AND pain)
2	TITLE-ABS-KEY (hospitalisation)
3	TITLE-ABS-KEY (cost AND of AND illness)
4	TITLE-ABS-KEY (absenteeism)
5	TITLE-ABS-KEY (ambulatory AND care)
6	TITLE-ABS-KEY (drug AND costs)
7	TITLE-ABS-KEY (emergency AND medical AND services)
8	TITLE-ABS-KEY (healthcare AND costs)
9	TITLE-ABS-KEY (nursing AND services)
10	TITLE-ABS-KEY (economics)
11	TITLE-ABS-KEY (physicians)
12	TITLE-ABS-KEY (burden)
13	TITLE-ABS-KEY (clinical AND impact)
14	TITLE-ABS-KEY (utilization)
15	TITLE-ABS-KEY (burden AND of AND illness)
16	TITLE-ABS-KEY (cost)
17	TITLE-ABS-KEY (nursing AND cost)
18	TITLE-ABS-KEY (physician AND cost)
19	TITLE-ABS-KEY (physician AND visit)
20	(TITLE-ABS-KEY (hospitalisation)) OR (TITLE-ABS-KEY (cost AND of AND illness)) OR (TITLE-ABS-KEY (absenteeism)) OR (TITLE-ABS-KEY (ambulatory AND care)) OR (TITLE-ABS-KEY (drug AND costs)) OR (TITLE-ABS-KEY (emergency AND medical AND services)) OR (TITLE-ABS-KEY (healthcare AND costs)) OR (TITLE-ABS-KEY (nursing AND services)) OR (TITLE-ABS-KEY (economics)) OR (TITLE-ABS-KEY (physicians)) OR (TITLE-ABS-KEY (burden)) OR (TITLE-ABS-KEY (clinical AND impact)) OR (TITLE-ABS-KEY (utilization)) OR (TITLE-ABS-KEY (burden AND of AND illness)) OR (TITLE-ABS-KEY (cost)) OR (TITLE-ABS-KEY (nursing AND cost)) OR (TITLE-ABS-KEY (physician AND cost)) OR (TITLE-ABS-KEY (physician AND visit))
21	(TITLE-ABS-KEY (low AND back AND pain)) AND ((TITLE-ABS-KEY (hospitalisation)) OR (TITLE-ABS-KEY (cost AND of AND illness)) OR (TITLE-ABS-KEY (absenteeism)) OR (TITLE-ABS-KEY (ambulatory AND care)) OR (TITLE-ABS-KEY (drug AND costs)) OR (TITLE-ABS-KEY (emergency AND medical AND services)) OR (TITLE-ABS-KEY (healthcare AND costs)) OR (TITLE-ABS-KEY (nursing AND services)) OR (TITLE-ABS-KEY (economics)) OR (TITLE-ABS-KEY (physicians)) OR (TITLE-ABS-KEY (burden)) OR (TITLE-ABS-KEY (clinical AND impact)) OR (TITLE-ABS-KEY (utilization)) OR (TITLE-ABS-KEY (burden AND of AND illness)) OR (TITLE-ABS-KEY (cost)) OR (TITLE-ABS-KEY (nursing AND cost)) OR (TITLE-ABS-KEY (physician AND cost)) OR (TITLE-ABS-KEY (physician AND visit)))

Appendix 1c: PubMed (inception to 10th December/2021

Query	Search Details
((((((((((((((((Hospitalisation[Title/Abstract] AND ((humans[Filter]) AND (english[Filter]))) OR (cost of illness[Title/Abstract] AND ((humans[Filter]) AND (english[Filter])))) OR (Absenteeism[Title/Abstract] AND ((humans[Filter]) AND (english[Filter])))) OR (Ambulatory care[Title/Abstract] AND ((humans[Filter]) AND (english[Filter])))) OR (Drug costs[Title/Abstract] AND ((humans[Filter]) AND (english[Filter])))) OR (Emergency medical services[Title/Abstract] AND ((humans[Filter]) AND (english[Filter])))) OR (healthcare costs[Title/Abstract] AND ((humans[Filter]) AND (english[Filter])))) OR (nursing services[Title/Abstract] AND ((humans[Filter]) AND (english[Filter])))) OR (economics[Title/Abstract] AND ((humans[Filter]) AND (english[Filter])))) OR (physicians[Title/Abstract] AND ((humans[Filter]) AND (english[Filter])))) OR (burden[Title/Abstract] AND ((humans[Filter]) AND (english[Filter])))) OR (clinical impact[Title/Abstract] AND ((humans[Filter]) AND (english[Filter])))) OR (Utilization[Title/Abstract] AND ((humans[Filter]) AND (english[Filter])))) OR (burden of illness[Title/Abstract] AND ((humans[Filter]) AND (english[Filter])))) OR (cost[Title/Abstract] AND ((humans[Filter]) AND (english[Filter])))) OR (nursing cost[Title/Abstract] AND ((humans[Filter]) AND (english[Filter])))) OR (physician visit[Title/Abstract] AND ((humans[Filter]) AND (english[Filter])))) AND (low back pain[Title/Abstract] AND ((humans[Filter]) AND (english[Filter])))	((("Hospitalisation"[Title/Abstract] AND ("humans"[MeSH Terms] AND "english"[Language])) OR ("cost of illness"[Title/Abstract] AND ("humans"[MeSH Terms] AND "english"[Language])) OR ("Absenteeism"[Title/Abstract] AND ("humans"[MeSH Terms] AND "english"[Language])) OR ("ambulatory care"[Title/Abstract] AND ("humans"[MeSH Terms] AND "english"[Language])) OR ("drug costs"[Title/Abstract] AND ("humans"[MeSH Terms] AND "english"[Language])) OR ("emergency medical services"[Title/Abstract] AND ("humans"[MeSH Terms] AND "english"[Language])) OR ("healthcare costs"[Title/Abstract] AND ("humans"[MeSH Terms] AND "english"[Language])) OR ("nursing services"[Title/Abstract] AND ("humans"[MeSH Terms] AND "english"[Language])) OR ("economics"[Title/Abstract] AND ("humans"[MeSH Terms] AND "english"[Language])) OR ("physicians"[Title/Abstract] AND ("humans"[MeSH Terms] AND "english"[Language])) OR ("burden"[Title/Abstract] AND ("humans"[MeSH Terms] AND "english"[Language])) OR ("clinical impact"[Title/Abstract] AND ("humans"[MeSH Terms] AND "english"[Language])) OR ("Utilization"[Title/Abstract] AND ("humans"[MeSH Terms] AND "english"[Language])) OR ("burden of illness"[Title/Abstract] AND ("humans"[MeSH Terms] AND "english"[Language])) OR ("cost"[Title/Abstract] AND ("humans"[MeSH Terms] AND "english"[Language])) OR ("nursing cost"[Title/Abstract] AND ("humans"[MeSH Terms] AND "english"[Language])) OR ("physician visit"[Title/Abstract] AND ("humans"[MeSH Terms] AND "english"[Language]))) AND ("low back pain"[Title/Abstract] AND ("humans"[MeSH Terms] AND "english"[Language]))) AND ((humans[Filter]) AND (english[Filter]))
low back pain[Title/Abstract]	("low back pain"[Title/Abstract]) AND ((humans[Filter]) AND (english[Filter]))
physician visit[Title/Abstract]	("physician visit"[Title/Abstract]) AND ((humans[Filter]) AND (english[Filter]))
nursing cost[Title/Abstract]	("nursing cost"[Title/Abstract]) AND ((humans[Filter]) AND (english[Filter]))
cost[Title/Abstract]	("cost"[Title/Abstract]) AND ((humans[Filter]) AND (english[Filter]))
burden of illness[Title/Abstract]	("burden of illness"[Title/Abstract]) AND ((humans[Filter]) AND (english[Filter]))
utilization[Title/Abstract]	("Utilization"[Title/Abstract]) AND ((humans[Filter]) AND (english[Filter]))
clinical impact[Title/Abstract]	("clinical impact"[Title/Abstract]) AND ((humans[Filter]) AND (english[Filter]))
burden[Title/Abstract]	("burden"[Title/Abstract]) AND ((humans[Filter]) AND (english[Filter]))
physicians[Title/Abstract]	("physicians"[Title/Abstract]) AND ((humans[Filter]) AND (english[Filter]))
economics[Title/Abstract]	("economics"[Title/Abstract]) AND ((humans[Filter]) AND (english[Filter]))
nursing services[Title/Abstract]	("nursing services"[Title/Abstract]) AND ((humans[Filter]) AND (english[Filter]))
healthcare costs[Title/Abstract]	("healthcare costs"[Title/Abstract]) AND ((humans[Filter]) AND (english[Filter]))
emergency medical services[Title/Abstract]	("emergency medical services"[Title/Abstract]) AND ((humans[Filter]) AND (english[Filter]))
Drug costs[Title/Abstract]	("drug costs"[Title/Abstract]) AND ((humans[Filter]) AND (english[Filter]))
Ambulatory care[Title/Abstract]	("ambulatory care"[Title/Abstract]) AND ((humans[Filter]) AND (english[Filter]))
Absenteeism[Title/Abstract]	("Absenteeism"[Title/Abstract]) AND ((humans[Filter]) AND (english[Filter]))
cost of illness[Title/Abstract]	("cost of illness"[Title/Abstract]) AND ((humans[Filter]) AND (english[Filter]))
hospitalisation[Title/Abstract]	("Hospitalisation"[Title/Abstract]) AND ((humans[Filter]) AND (english[Filter]))

Appendix 2: List of excluded studies

Study	Country	Design	Aim	Justification for exclusion
Safiri et al., 2021	Global	Model	To determine the burden of other musculoskeletal disorders between 1990 and 2017 at the global, regional and national level across 195 countries and territories	Estimate not for low back pain
Barreto & Sá, 2019	Brazil	Questionnaire	To estimate indirect cost related to chronic pain from an employer's perspective	Estimate not for low back pain
Du et al., 2019	China	CR	To systematically explore the correlates of emotional distress in patients with chronic low back pain	Not clinical or economic burden study.
Driscoll et al., 2014	Global	Servery	To quantify the burden arising from low back pain (LBP) due to occupational exposure to ergonomic risk factors.	Not clinical or economic burden study.
Hoy et al., 2014	Global	Survey	To estimate the global burden of LBP.	Not related to the research topic
Smith et al., 2014	Global	Systematic reviews	To estimate disability from the remainder of musculoskeletal (MSK) disorders	Not clinical or economic burden study
Vos et al., 2012	Worldwide	Model	To describe the approach to undertaking past burden of disease assessments with the available evidence.	Not related
Moradi-Lakeh et al., 2017	EMR	Systematic analysis	To report the burden of musculoskeletal disorders in the Eastern Mediterranean Region.	Not clinical or economic burden study
de David et al., 2020	Brazil	Model	To describe the current status and trends of the burden due to LBP in Brazil	Prevalence and incidence
Amaefule et al., 2021	Nigeria	CR	To estimate the burden of chronic musculoskeletal Disorders amongst patients attending orthopaedic outpatients' clinic	Ful article not available
Aminde et al., 2020	Cameroon	CR	To evaluate health-related quality of life (HRQoL) and its determinants in chronic low back pain patients in Cameroon.	Not clinical or economic burden study
Dagenais et al., 2008	USA	CR	To conduct a systematic review of LBP cost of illness studies	A systematic review
Wu et al., 2019	China	CR	To quantify the prevalence and years lived with disability (YLDs) caused by LBP in China	Not clinical or economic burden study
Dienye et al., 2016	Nigeria	CR	To determine the prevalence of LBP	Prevalence and incidence
Galukande et al., 2006	Uganda	CR	To assess and document the disability associated with low back pain in terms of sick leave	Not clinical or economic burden study
Faezi et al., 2020	Iran	Interviews	To evaluate health-care access and utilization among patients with LBP in Iran	Not clinical or economic burden study
Igwesi-Chidobe et al., 2019	Nigeria	Questionnaire	To assess self-reported disability in a low-literate population with chronic LBP	Not clinical or economic burden study
Michalik et al., 2015	Poland	CR	To present the epidemiology data on back pain in Poland	Prevalence and incidence
Carregaro et al., 2019	Brazil	Prevalence-based	To estimate the direct healthcare costs of spinal disorders in Brazil over 2016.	Not low back pain
Kisa et al., 2020	Global	Systematic analysis	To provides an overview of the influence of occupational risk factors on the global burden of disease	Not low back pain
Maetzel & Li, 2002	N/A	Systematic review	To gain a better understanding of the societal costs of LBP	A review of studies

EMR = Eastern Mediterranean Region; CR = Cross sectional
