# Supplementary material for: Clinical and economic burden of low back pain in low- and middle-income countries: a systematic review
Source: BMJ Open. 2023 Apr 25;13(4):e064119. doi: 10.1136/bmjopen-2022-064119 (PMC10151982; doi:10.1136/bmjopen-2022-064119)
Supplement: Author's manuscript [file bmjopen-2022-064119.draft_revisions.pdf]

BMJ Open

BMJ Open is committed to open peer review. As part of this commitment we make the peer review history of every article we publish publicly available.

When an article is published we post the peer reviewers' comments and the authors' responses online. We also post the versions of the paper that were used during peer review. These are the versions that the peer review comments apply to.

The versions of the paper that follow are the versions that were submitted during the peer review process. They are not the versions of record or the final published versions. They should not be cited or distributed as the published version of this manuscript.

BMJ Open is an open access journal and the full, final, typeset and author-corrected version of record of the manuscript is available on our site with no access controls, subscription charges or pay-per-view fees (http://bmjopen.bmj.com).

If you have any questions on BMJ Open's open peer review process please email info.bmjopen@bmj.com

BMJ Open

Clinical and economic burden of low back pain in low- and middle-income countries: a systematic review

Journal:	*BMJ Open*
Manuscript ID	bmjopen-2022-064119
Article Type:	Original research
Date Submitted by the Author:	25-Apr-2022
Complete List of Authors:	Fatoye, Francis; Manchester Metropolitan University<br>Gebrye, Tadesse; Manchester Metropolitan University<br>Mbada, Chidozie Emmanuel; Obafemi Awolowo University, Department of Medical Rehabilitation<br>Useh, Ushotanefe; North West Univ,
Keywords:	Health economics < HEALTH SERVICES ADMINISTRATION & MANAGEMENT, PAIN MANAGEMENT, Spine < ORTHOPAEDIC & TRAUMA SURGERY, Back pain < ORTHOPAEDIC & TRAUMA SURGERY

SCHOLARONE™
Manuscripts

I, the Submitting Author has the right to grant and does grant on behalf of all authors of the Work (as defined in the below author licence), an exclusive licence and/or a non-exclusive licence for contributions from authors who are: i) UK Crown employees; ii) where BMJ has agreed a CC-BY licence shall apply, and/or iii) in accordance with the terms applicable for US Federal Government officers or employees acting as part of their official duties; on a worldwide, perpetual, irrevocable, royalty-free basis to BMJ Publishing Group Ltd ("BMJ") its licensees and where the relevant Journal is co-owned by BMJ to the co-owners of the Journal, to publish the Work in this journal and any other BMJ products and to exploit all rights, as set out in our licence.

The Submitting Author accepts and understands that any supply made under these terms is made by BMJ to the Submitting Author unless you are acting as an employee on behalf of your employer or a postgraduate student of an affiliated institution which is paying any applicable article publishing charge ("APC") for Open Access articles. Where the Submitting Author wishes to make the Work available on an Open Access basis (and intends to pay the relevant APC), the terms of reuse of such Open Access shall be governed by a Creative Commons licence – details of these licences and which Creative Commons licence will apply to this Work are set out in our licence referred to above.

Other than as permitted in any relevant BMJ Author's Self Archiving Policies, I confirm this Work has not been accepted for publication elsewhere, is not being considered for publication elsewhere and does not duplicate material already published. I confirm all authors consent to publication of this Work and authorise the granting of this licence.

For peer review only

Clinical and economic burden of low back pain in low- and middle-income countries: a systematic review

Francis Fatoye[1, 3], Tadesse Gebrye[1], Chidozie Mbada [2], Ushotanefe Useh [3]

[1] Department of Health Professions, Manchester Metropolitan University, Birley Fields Campus, Bonsall Street, Manchester, M156GX.

[2] Department of Medical Rehabilitation, College of Health Sciences, Obafemi Awolowo University, Ile-Ife, Nigeria.

[3] Lifestyle Diseases, Faculty of Health Sciences, North-West University, South Africa

Correspondence author: Professor Francis Fatoye,

Department of Health Professions,

Faculty of health, Psychology, and Social Care,

Manchester Metropolitan University, Brooks Building,

53 Bonsall Street, Manchester UK, M15 6GX.

Phone: 44 (0) 161 247 2963

Email: t.gebrye@mmu.ac.uk

For peer review only

Abstract

Objectives: Low back pain (LBP) is a leading cause of disability, work absenteeism globally, and it poses significant clinical and economic burden. This study aimed to synthesise the clinical and economic burden of LBP in Low- and Middle-Income Countries (LMICs).

Methods: A systematic review following the Preferred Reporting Items for Systematic Reviews and Meta-Analyses guidelines was performed. PubMed, Medline, Cinhal, PsycINFO, AMED, Embase and Scopus databases were systematically searched for studies that examined the clinical and economic burden of LBP in LMICs, published from inception to December 10, 2021. Only studies with clearly stated methodologies and published in English were eligible for review.

Results: Nine studies met the inclusion criteria. Of these, three of them were clinical burden studies. The mean Newcastle–Ottawa quality assessment scale (NOS) score of the included studies was 4, with an average from 3 to 6. The included studies were conducted in Argentina, Brazil, China, Ethiopia, Nigeria, and Republic of Serbia. The review highlighted that there was a significant clinical burden of LBP, with high rates of hospitalisation in LMICs. Due to variation of methodological approaches, the reported cost estimates were inconsistent across the studies. A US$2.2 billion and US$1226.25 annual costs of LBP per population and patient level, respectively were reported. Considerable indirect costs were also reported mainly due to work absenteeism.

Conclusion: This is the first systematic literature review to suggest that LBP is associated with significantly high rates of hospitalisation and work absenteeism. As LBP is an important threat to the population, health professionals and policymakers are to put in place appropriate programmes to reduce the clinical and economic burden associated with LBP in LMICs.

Keywords: Low back pain, economic, clinical, LMICs

For peer review only

Introduction

Low-back pain (LBP) is an important health problem and covers radicular, axial lumbosacral, and referred pain [1]. The lifetime, 1-year and point prevalence of LBP worldwide ranged from 11% to 84%, 22% to 65% and 12% to 33%, respectively [2]. The 1-year incidence of people who have a first-ever episode and any episode of LBP ranged 6.5% to 15.4% and 1.5% to 36% [3]. LBP is associated with increased health care costs, length of stay in hospitals, morbidity and mortality in both high- and low-income countries [4].

To reduce the clinical and economic burden associated with LBP, it is important that prevention and management strategies of LBP are implemented. Prevention and management strategies are aimed at controlling pain, maintaining function and prevention of exacerbation of LBP [5]. The American College of Physicians and the American Pain Society recommends that individual with LBP should remain active, and they provide information about effective self-care options, use of medications with proven benefits, interdisciplinary rehabilitation, exercise, acupuncture, massage, spinal manipulation, yoga, cognitive behavioural therapy, or relaxation for the treatment of LBP [6].

The implementation of these treatments remains a challenge for clinical practice and research [7]. Because of this challenge, LBP was listed among the top 10 diseases and injuries that account for the highest number of disability-adjusted life years (DALYs) worldwide [8]. The direct medical and indirect costs of LBP are more than $50 billion per annum and could be as high as $100 billion at the extreme [9]. Evidence suggests that as much as 80% to 90% of the populations' work in low- and middle-income countries (LMICs) entails heavy labour such as carrying heavy loads on the back or head [10]. The risk factors of LBP include low educational status, stress, anxiety, depression, job dissatisfaction, low levels of social support in the workplace and whole-body vibration [3]. The causes and risk factors for LBP in LMIC is substantial [11]. Thus, the likelihood of having LBP is higher in LMICs than high income countries.

Many studies have been published evaluating the clinical and economic burden of LBP in LMICs. However, to date there are no studies that have summarised literature on the clinical and economic burden of LBP in LMICs. Therefore, this systematic review was aimed to evaluated and synthesised literature regarding the clinical and economic burden of LBP in LMICs.

Methods

Search protocol and registration

In this study, we used the Preferred Reporting Items for Systematic Reviews and Meta-Analysis guideline. A protocol for this systematic review was prospectively registered on PROSPERO and can be found at https://www.crd.york.ac.uk/prospero/#recordDetails ID = CRD42020196335.

For peer review only - http://bmjopen.bmj.com/site/about/guidelines.xhtml

Search strategy

The systematic review was carried out in the PubMed, Medline, CINAHL, PsycINFO, AMED, Embase and Scopus databases with studies published from inception to December 10, 2021.The following keywords were used in the search: low back pain, hospitalisation, cost of illness, absenteeism, ambulatory care, drug costs, emergency medical services, healthcare costs, nursing services, economics, physicians visit, clinical impact, utilization, burden of illness, cost, nursing cost. These search terms were combined using conjunctions words 'AND' or 'OR'. Further, a manual search of reference sections of the included studies was also checked for additional studies. The search was performed independently by two authors (TG & FF) to avoid the presence of bias in the selection and exclusion of studies. Any disagreement was resolved by discussion with the third the author (CEM).

Inclusion and exclusion criteria

Included in this review were studies among patients with LBP; original research findings related to costs (direct and indirect) in LMIC as defined by World Health Organization (WHO); the contexts of interest were hospitals, primary healthcare clinics and home settings; observational (cross-sectional or surveys), randomised controlled trials (RCTs) and modelling studies and published in peer-reviewed journals. Review articles, editorials, letters to the editor, news reports, conference abstracts, comments, as well as the results of dissertations were excluded.

Study selection and assessment of methodological quality

After selection of the articles by the search strategy in each database, duplicate articles were excluded. Following the removal of duplicates, titles and abstracts were screened by two reviewers (TG & FF) to identify eligible studies. The full texts of the identified studies were checked against the inclusion and exclusion criteria. Discrepancies for study selection were resolved through discussion with other investigators (CEM & UU). Having retrieved the full text of studies that met the inclusion criteria, they were assessed for methodological quality using the Newcastle–Ottawa quality assessment scale (NOS) for cohort studies [12]. The NOS contains nine items, categorized into three dimensions including selection and comparability. Studies were scored using a scale with a possible maximum of nine points where a score ≥ 6 indicated high-quality studies, a score between 3 - 6 as moderate and a score ≤ 3 as low quality.

Data extraction

Data were extracted by two independent review authors. The following information was extracted for each study: authors, country and year of publication, study objective, data source, inclusion criteria, LBP definition, population characteristics (size, % male, and mean age), hospitalisation, average total annual cost per patient and annual population cost. A summary table was used to display the extracted data. When there was disagreement, it was addressed through consultation with the third reviewer (CEM).

For peer review only - http://bmjopen.bmj.com/site/about/guidelines.xhtml

Data synthesis

A narrative synthesis of the extracted data was employed. Extracted data were summarized in evidence tables on a study-by-study. Because of the heterogeneity of study designs, interventions, follow-up and outcomes, no attempt was made to pool the results. If necessary, costs were converted to US$ using Salary converter [13]. Most of costs have been converted to US$ using purchasing power party and then inflation adjustments were calculated using the suggested country specific inflation index at [14]. Conversions were performed in February 2022.

Patient and public involvement

Patients and the public were not involved in the design or planning of the study.

Results

This systematic review generated 3,074 records in Scopus (n = 51), PubMed (n = 2,036), Embase (n = 700) and Medline, AMED, CINAHL, PsycINFO (n = 287) (Figure 1). Of these, 278 were duplicates. Following screening by titles and abstracts 2,766 studies were excluded, leaving 30 articles for further full text review. After reading the full text only 9 met the inclusion criteria and were eligible for analysis. The mean NOS score of the included studies was 4, with an average from 3 to 6 (Table 1). The NOS score showed all the included studies do not show comparability of cohorts on the basis of age, sex the design, and analysis.

....................Figure 1 about here.................

Characteristics of the included studies

The characteristics of the included studies are presented in Table 1. The clinical and economic burden of LBP studies were conducted in Nigeria (n = 3), Ethiopia (n = 1), Brazil (n = 2), China (n = 1), Republic of Serbia (n = 1), and Argentina (n = 1). Of the 9 eligible studies included in our review, 3 studies [15, 16, 17] reported the clinical impact of LBP in LMICs. Whereas the remaining studies reported the economic burden of LBP. The data sources of the included studies were interviews, questionnaire, population based cross-sectional study and medical records. Except two studies [16, 18], all the included studies reported LBP definition.

For peer review only

Table 1: Characteristics of clinical and economic burden studies included in the review

Reference/ Country	Data source	Study objective	Inclusion criteria	LBP definition	NOS/9
Beyera et al., [15]/ Ethiopia	Interview	To analyse hospital admission and associated factors	Individuals with LBP of ≥ 18 years of age. #Presented to health care facility for LBP in the past 1-year	Not provided	3
Carregaro et al., [16]/ Brazil	National databases, considering the period 2012–2016.	To estimate the healthcare expenditure and productivity losses related to LBP	≥ 19 years	ICD-10 codes	6
Laffont et al. [17]/ Argentina	The Statistics and Information Department of the Ministry of Health	To analyse the impact of LBP on hospitalisations between 2006 and 2010.	Either adult LBP or lumbosciatica (≥ 18 years)	ICD-10 codes	6
Odole et al. [18/ Nigeria	Questionnaire	To determine the economic burden of LBP patients in Ibadan	Individuals with LBP receiving physiotherapy in Ibadan	Not provided	3
Bello & Muhammad [19] /Nigeria	Questionnaire	To estimate the cost of LBP management	Patients with LBP, allow them to carry out activities for daily living even under difficulties.	ICD-9 codes	3
do Socorro et al., [20]/Brazil	Questionnaire	To assess resource utilization in the diagnosis, management, and hospitalization of patients	Participants were rheumatologists attending a national rheumatology medical congress.	Not provided	4
Birabi et al. [21]/ Nigeria	Medical records of Hospitals (220 patients)	To report the results of a retrospective study on the direct cost burden of LBP between 2010-2013	Patients whose care commenced from acute care stages, admitted where necessary.	ICD-10 codes	5
Lin et al. [22]/ Republic of China	Interview	To estimate the 12- month economic cost of LBP among female (1990 to 1991)	Nurses who had experienced LBP within the 12-month period	ICD-10 codes	3
Radoičlc et al. [23]/ Republic of Serbia	Interview (April 2016 to April 2017).	To estimate the cost of LBP	Diagnosed as M54, which refers to lumbar pain (Dorsalgia)	ICD-10 codes	4

Note: ICD = International Classification of Diseases; NOS = Newcastle–Ottawa quality assessment scale

Hospitalisation

A total of 3 studies reported data on rates of hospitalisation for LBP (Table 2). The rates of hospitalisation ranged between 13.4% to 18.7%. The highest rate of hospitalisation (18.7%) for LBP was reported in Argentina [17].

For peer review only - http://bmjopen.bmj.com/site/about/guidelines.xhtml

Table 2: Rate of hospitalisation for LBP patients

Study	Number of participants	Year	Hospitalizations for LBP
Beyera et al. [15]	543 (M = 316; F = 205)	Between June and November 2018	14.4%, 95% CI 11.4-17.3, #With an average length of stay (LOS) 7.4 days, 95% CI 6.4-8.8
Carregaro et al. [16]	880,000 diagnostic images	2012 to 2016	13.4% (inpatient admissions)
Laffont et al. [17]	A total of 154,430 discharges in which patients of any age were hospitalised	2006 and 2010	18.7%, #With an average LOS in the hospital of 3.8 days
do Socorro et al. [20]	207 rheumatologists (M = 62%; F = 38%)	N/A	An average of 574 patients with LBP were seen per year.

Note: N/A = Not available; M = Male; F = Female

Economic burden

The economic burden of the included studies is provided in Table 3. A total of 6 studies reported average annual cost per patient [15, 18, 19, 21, 22, 23]. One of the included studies reported annual cost per population [17]. Patients with the age range up to 85 years were included in the included studies. Due to variation in methodological approaches, the reported cost estimates were inconsistent across the studies. However, up to an average of annual costs of US$2.2 billion and US$1226.25 per population and per patient level, respectively were reported. Up to $1.7 billion indirect costs were also reported mainly due to work absenteeism [17].

Table 3: Summary of studies that reported patient and population -level total costs for LBP

Reference	Age group (years)	Average annual cost per patient	Inflated 2022 $US
Carregaro et al. [16]	≥ 19	Total costs = US$ 2.2 billion¶ Indirect cost = 1.7 billion ¶ (productivity losses)	Total cost = US$2.5 billion¶
Odole et al. [18]	30 to 80	Total cost = US$1125.27 ± 623.39 Direct costs = US$927.20 ± 600.26. Indirect cost = US$198.08 ± 136.15	Total cost = US$5047.5 ± 2796.3 Direct costs = US$4158.1 ± 2691); Indirect cost = US$888 ± 610.
Bello & Muhammad, [19]	30 to 70	Total = US$763.27 ± 543.97; Direct cost = US$647.28 ± 438.99; Indirect cost = US$115.99 ± 104.99	Total = US$ 3029.9 ± 2159.1 Direct cost = US$2569.5 ± 1742.6 Indirect cost = 460.1 ± 416.5
do Socorro et al. [20]	N/A	30% of patients with LBP would be absent from their work due to the condition.	N/A
Birabi et al. [21]	26 and 65	Total cost = US$1226.25 (government hospitals); US$4884.37 (private hospitals)	#US$3192.53 (government hospitals) #US$12716.41(private hospitals)
Lin et al. [22]	Mean age = 32.2 ± 9.2	Total costs = US$994.4 to US$1406.4.	N/A
Radoičlc et al. [23]	Median, 53.59 (28 – 85)	Total costs = €200.40 ± €86.65, Direct costs = €9.39 ± €6.66. Indirect costs = €182.00 ± €78.66	Total cost = €432.86 ± €187.2 Total indirect costs = €393.12 ± €169.99

Note: ¶ = per population; N/A = Not available

For peer review only - http://bmjopen.bmj.com/site/about/guidelines.xhtml

Discussion

To our knowledge this is the first systematic review to analyse the clinical and economic impact of LBP in LMICs. A total of 9 studies from six LMICs were included. Our review indicates that the clinical and economic burden of LBP is substantial in LMICs. The rate of hospitalisation ranged between 13.4% and 18.7% of patients with LBP. The findings of the review also suggest that LBP is associated with an annual cost of US$2.2 billion per population in LMICs. A clearer understanding of the clinical and economic burden of LBP in LMICs is therefore important for policy makers that they may consider appropriate prevention and management strategies for LBP.

Rate of hospitalisation of female patients with LBP was higher (53.7%) than their male counterpart [20]. The reason for this could be epidemiological where the proportion of LBP in female is in a higher proportion than males [24]. The average length of stay in hospital due to LBP in Argentina and Ethiopia was 3.8 days [20] and 7.4 days [16], respectively. The length of stay in hospital is directly associated with high financial impact of LBP, this is because of its healthcare and social costs [25].

We collected evidence of both direct and indirect economic burden of LBP. The overall burden varied based on characteristics of patient populations, geographic region and the methodologies adopted by the studies, this includes the individual patient or the population level perspective. For example, the societal annual costs amounted to US$ 2.2 billion per population where the productivity losses represented 79% of the costs reported in Brazil [17]. Overall, the considerable clinical and economic burden of LBP in LMICs could be due to its poorly equipped health and social system [26].

There are certain strength and limitations to this study. The main strength of this review is the comprehensiveness of the search terms, screening of numerous data bases, and assessment of methodological quality of the studies. Only English language studies were included. Therefore, it is possible that relevant literature published in other languages may have been excluded. it was not possible to undertake a meta-analysis for most of the included studies due to the adoption of different time horizons, different clinical outcomes and lack of data regarding mean and SD for costs [27]. Further, the quality of the included studies indicates that there is a need to interpret the clinical and economic burden results with caution.

In conclusion: We found that LBP is associated with significantly high rate of hospitalisation with a clear impact on length of stay in hospital and treatment costs. The findings of our analysis can be used by clinicians, and policymakers to better allocate resources for prevention and management strategies for LBP improve health outcomes and reduce the substantial burden associated with the condition. We also hope our results will be of use to researchers planning to evaluate the cost-effectiveness of various strategies for preventing LBP in LMICs.

Authors contribution: F.F. & T.G. performed electronic searches and assessed title and abstract. C.EM. and U.U. were consulted in case of disagreement. F.F. & T.G. performed the methodological quality assessment and extracted the data. T.G. & C.EM. wrote the original draft preparation. F.F. & U.U. reviewed, revised, and edited this paper. All authors read and approved the final draft of the manuscript**.**

For peer review only - http://bmjopen.bmj.com/site/about/guidelines.xhtml

Ethical approval: For this study ethical approval is not required.

Competing interest: The authors have no competing interests to declare.

Funding information: This research did not receive any specific grant from funding agencies in the public, commercial, or not-for-profit sectors.

Informed consent: The patient's written informed consent was not made, as this was a systematic review study.

Data sharing: All data related to this work are available in this research article.

For peer review only

For peer review only - http://bmjopen.bmj.com/site/about/guidelines.xhtml

References

1. Urits I, Burshtein A, Sharma M, Testa L, Gold PA, Orhurhu V, Viswanath O, Jones MR, Sidransky MA, Spektor B, Kaye AD. Low back pain, a comprehensive review: pathophysiology, diagnosis, and treatment. Current pain and headache reports. 2019 Mar;23(3):1-0.

2. Tay M, Sian SC, Eow CZ, Ho KL, Ong JH, Sirisena D. Ultrasound-Guided Lumbar Spine Injection for Axial and Radicular Pain: A Single Institution Early Experience. Asian Spine Journal. 2021 Apr;15(2):216.

3. Hoy D, Brooks P, Blyth F, Buchbinder R. The epidemiology of low back pain. Best practice & research Clinical rheumatology. 2010 Dec 1;24(6):769-81.

4. Dagenais S, Caro J, Haldeman S. A systematic review of low back pain cost of illness studies in the United States and internationally. The spine journal. 2008 Jan 1;8(1):8-20.

5. Kuritzky L, Samraj GP. Nonsteroidal anti-inflammatory drugs in the treatment of low back pain. Journal of Pain Research. 2012;5:579.

6. Chou R, Qaseem A, Snow V, Casey D, Cross Jr JT, Shekelle P, Owens DK, Clinical Efficacy Assessment Subcommittee of the American College of Physicians and the American College of Physicians/American Pain Society Low Back Pain Guidelines Panel*. Diagnosis and treatment of low back pain: a joint clinical practice guideline from the American College of Physicians and the American Pain Society. Annals of internal medicine. 2007 Oct 2;147(7):478-91.

7. Koes BW, Van Tulder M, Lin CW, Macedo LG, McAuley J, Maher C. An updated overview of clinical guidelines for the management of non-specific low back pain in primary care. European Spine Journal. 2010 Dec;19(12):2075-94.

8. Vos T, Flaxman AD, Naghavi M, Lozano R, Michaud C, Ezzati M, Shibuya K, Salomon JA, Abdalla S, Aboyans V, Abraham J. Years lived with disability (YLDs) for 1160 sequelae of 289 diseases and injuries 1990–2010: a systematic analysis for the Global Burden of Disease Study 2010. The lancet. 2012 Dec 15;380(9859):2163-96.

9. Hoy D, March L, Brooks P, Blyth F, Woolf A, Bain C, Williams G, Smith E, Vos T, Barendregt J, Murray C. The global burden of low back pain: estimates from the Global Burden of Disease 2010 study. Annals of the rheumatic diseases. 2014 Jun 1;73(6):968-74.

10. Louw QA, Morris LD, Grimmer-Somers K. The prevalence of low back pain in Africa: a systematic review. BMC Musculoskeletal disorders. 2007 Dec;8(1):1-4.

11. Stewart Williams J, Ng N, Peltzer K, Yawson A, Biritwum R, Maximova T, Wu F, Arokiasamy P, Kowal P, Chatterji S. Risk factors and disability associated with low back pain in older adults in low-and middle-

For peer review only - http://bmjopen.bmj.com/site/about/guidelines.xhtml

For peer review only

income countries. Results from the WHO Study on Global AGEing and Adult Health (SAGE). PloS one. 2015 Jun 4;10(6):e0127880.

12. Stang A. Critical evaluation of the Newcastle-Ottawa scale for the assessment of the quality of nonrandomized studies in meta-analyses. European journal of epidemiology. 2010 Sep;25(9):603-5.
13. Salary converter. Purchasing power parities. 2017 http:// salaryconverter. nigelb. me/ (accessed February 2022).
14. World Bank. World Bank open data. 2022. Available at: https://data.worldbank.org/indicator/FP.CPI. TOTL. Accessed February 2022.
15. Bello B, Muhammad IT. Estimation of economic cost of low back pain management in Kano, Nigeria. Adv Life Sci Med. 2017;3:01-8.
16. Beyera GK, O'Brien J, Campbell S. Hospital admission and associated factors among individuals presenting to healthcare facilities for low back pain in Ethiopia. International Journal of Rheumatic Diseases. 2020 Jun;23(6):763-71.
17. Carregaro RL, Tottoli CR, Rodrigues DD, Bosmans JE, da Silva EN, van Tulder M. Low back pain should be considered a health and research priority in Brazil: Lost productivity and healthcare costs between 2012 to 2016. PloS one. 2020 Apr 1;15(4):e0230902.
18. do Socorro Margarido M, Kowalski SC, Natour J, Ferraz MB. Acute low back pain: diagnostic and therapeutic practices reported by Brazilian rheumatologists. Spine. 2005 Mar 1;30(5):567-71.
19. Birabi, BN, Oke, KI, Dienye, PO, & Okafor, UAC. Cost Burden of Low Back Pain Amon Adults in Southern Nigeria. International Journal of Medical and Applied Science, 2014 June 4; 3 (3): 24-29.
20. Laffont M, Sequeira G, Kerzberg EM, Marconi E, Guevel C, de las Mercedes Fernández M. The non-silent epidemic: low back pain as a primary cause of hospitalisation. Rheumatology international. 2016 May;36(5):673-7.
21. Lin MR, Tsauo JY, Wang JD. Determinants of economic cost related to low back pain among nurses at a University Hospital. International Journal of Occupational and Environmental Health. 1996 Oct 1;2(4):257-63.
22. Radoičlc M, Božovic B, Ilić K, Jankovic S, AnĐelković J, Kostic M. Pharmacoeconomic aspects of low back pain treatment: Cost of illness study in the republic of Serbia. Acta Medica Portuguesa. 2019 Apr 1.
23. Odole AC, Akinpelu AA, Adekanla BA, Obisanya OB. Economic burden of low back pain on patients seen at the outpatient Physiotherapy clinics of secondary and tertiary health institutions in Ibadan. Journal of the Nigeria Society of Physiotherapy. 2011 Dec 28;18(1):43-8.

For peer review only - http://bmjopen.bmj.com/site/about/guidelines.xhtml

24. Hoy D, Bain C, Williams G, March L, Brooks P, Blyth F, Woolf A, Vos T, Buchbinder R. A systematic review of the global prevalence of low back pain. Arthritis & Rheumatism. 2012 Jun;64(6):2028-37.

25. Anema JR, Schellart AJ, Cassidy JD, Loisel P, Veerman TJ, Van der Beek AJ. Can cross country differences in return-to-work after chronic occupational back pain be explained? An exploratory analysis on disability policies in a six country cohort study. Journal of occupational rehabilitation. 2009 Dec;19(4):419-26.

26. Hartvigsen J, Hancock MJ, Kongsted A, Louw Q, Ferreira ML, Genevay S, Hoy D, Karppinen J, Pransky G, Sieper J, Smeets RJ. What low back pain is and why we need to pay attention. The Lancet. 2018 Jun 9;391(10137):2356-67.

27. Higgins MD, Green RJ, Leeson MS. Optical wireless for intravehicle communications: A channel viability analysis. IEEE Transactions on Vehicular Technology. 2011 Nov 18;61(1):123-9.

For peer review only

For peer review only - http://bmjopen.bmj.com/site/about/guidelines.xhtml

Figure

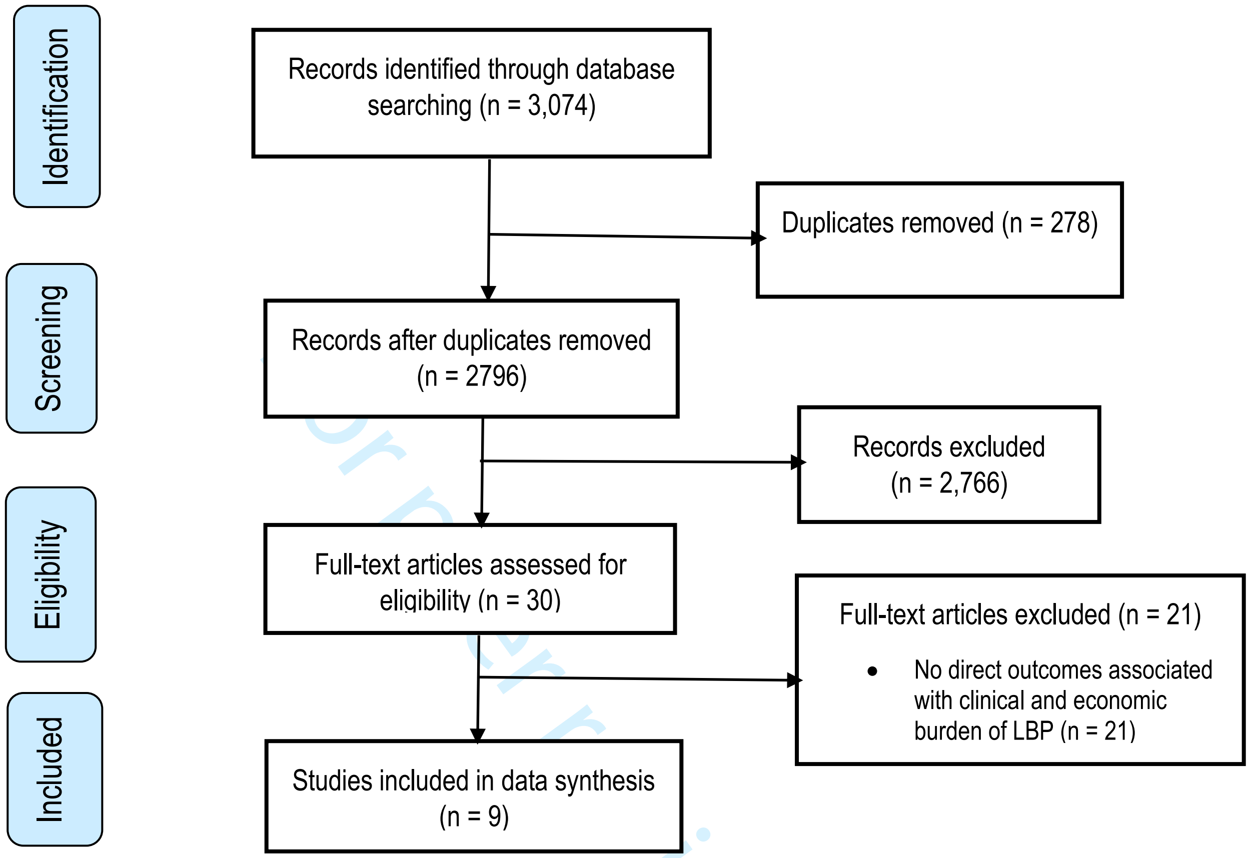

Figure 1. Flow diagram of publications included and excluded in the review

For peer review only

PRISMA 2020 Checklist

Section and Topic	Item #	Checklist item	Location where item is reported
**TITLE**			
Title	1	Identify the report as a systematic review.	1
**ABSTRACT**			
Abstract	2	See the PRISMA 2020 for Abstracts checklist.	2
**INTRODUCTION**			
Rationale	3	Describe the rationale for the review in the context of existing knowledge.	3
Objectives	4	Provide an explicit statement of the objective(s) or question(s) the review addresses.	3
**METHODS**			
Eligibility criteria	5	Specify the inclusion and exclusion criteria for the review and how studies were grouped for the syntheses.	4
Information sources	6	Specify all databases, registers, websites, organisations, reference lists and other sources searched or consulted to identify studies. Specify the date when each source was last searched or consulted.	4
Search strategy	7	Present the full search strategies for all databases, registers and websites, including any filters and limits used.	4
Selection process	8	Specify the methods used to decide whether a study met the inclusion criteria of the review, including how many reviewers screened each record and each report retrieved, whether they worked independently, and if applicable, details of automation tools used in the process.	4
Data collection process	9	Specify the methods used to collect data from reports, including how many reviewers collected data from each report, whether they worked independently, any processes for obtaining or confirming data from study investigators, and if applicable, details of automation tools used in the process.	4
Data items	10a	List and define all outcomes for which data were sought. Specify whether all results that were compatible with each outcome domain in each study were sought (e.g. for all measures, time points, analyses), and if not, the methods used to decide which results to collect.	5
	10b	List and define all other variables for which data were sought (e.g. participant and intervention characteristics, funding sources). Describe any assumptions made about any missing or unclear information.	5
Study risk of bias assessment	11	Specify the methods used to assess risk of bias in the included studies, including details of the tool(s) used, how many reviewers assessed each study and whether they worked independently, and if applicable, details of automation tools used in the process.	4
Effect measures	12	Specify for each outcome the effect measure(s) (e.g. risk ratio, mean difference) used in the synthesis or presentation of results.	N/R
Synthesis methods	13a	Describe the processes used to decide which studies were eligible for each synthesis (e.g. tabulating the study intervention characteristics and comparing against the planned groups for each synthesis (item #5)).	5
	13b	Describe any methods required to prepare the data for presentation or synthesis, such as handling of missing summary statistics, or data conversions.	5
	13c	Describe any methods used to tabulate or visually display results of individual studies and syntheses.	5
	13d	Describe any methods used to synthesize results and provide a rationale for the choice(s). If meta-analysis was performed, describe the model(s), method(s) to identify the presence and extent of statistical heterogeneity, and software package(s) used.	N/R
	13e	Describe any methods used to explore possible causes of heterogeneity among study results (e.g. subgroup analysis, meta-regression).	N/R
	13f	Describe any sensitivity analyses conducted to assess robustness of the synthesized results.	N/R
Reporting bias assessment	14	Describe any methods used to assess risk of bias due to missing results in a synthesis (arising from reporting biases).	5
Certainty	15	Describe any methods used to assess certainty (or confidence) in the body of evidence for an outcome.	N/R

For peer review only - http://bmjopen.bmj.com/site/about/guidelines.xhtml

For peer review only

PRISMA 2020 Checklist

Section and Topic	Item #	Checklist item	Location where item is reported
assessment			
**RESULTS**			
Study selection	16a	Describe the results of the search and selection process, from the number of records identified in the search to the number of studies included in the review, ideally using a flow diagram.	5
	16b	Cite studies that might appear to meet the inclusion criteria, but which were excluded, and explain why they were excluded.	5
Study characteristics	17	Cite each included study and present its characteristics.	6
Risk of bias in studies	18	Present assessments of risk of bias for each included study.	6
Results of individual studies	19	For all outcomes, present, for each study: (a) summary statistics for each group (where appropriate) and (b) an effect estimate and its precision (e.g. confidence/credible interval), ideally using structured tables or plots.	6
Results of syntheses	20a	For each synthesis, briefly summarise the characteristics and risk of bias among contributing studies.	6
	20b	Present results of all statistical syntheses conducted. If meta-analysis was done, present for each the summary estimate and its precision (e.g. confidence/credible interval) and measures of statistical heterogeneity. If comparing groups, describe the direction of the effect.	N/R
	20c	Present results of all investigations of possible causes of heterogeneity among study results.	N/R
	20d	Present results of all sensitivity analyses conducted to assess the robustness of the synthesized results.	N/R
Reporting biases	21	Present assessments of risk of bias due to missing results (arising from reporting biases) for each synthesis assessed.	N/R
Certainty of evidence	22	Present assessments of certainty (or confidence) in the body of evidence for each outcome assessed.	N/R
**DISCUSSION**			
Discussion	23a	Provide a general interpretation of the results in the context of other evidence.	8
	23b	Discuss any limitations of the evidence included in the review.	8
	23c	Discuss any limitations of the review processes used.	8
	23d	Discuss implications of the results for practice, policy, and future research.	8
**OTHER INFORMATION**			
Registration and protocol	24a	Provide registration information for the review, including register name and registration number, or state that the review was not registered.	3
	24b	Indicate where the review protocol can be accessed, or state that a protocol was not prepared.	3
	24c	Describe and explain any amendments to information provided at registration or in the protocol.	N/R
Support	25	Describe sources of financial or non-financial support for the review, and the role of the funders or sponsors in the review.	9
Competing interests	26	Declare any competing interests of review authors.	9
Availability of data, code and other materials	27	Report which of the following are publicly available and where they can be found: template data collection forms; data extracted from included studies; data used for all analyses; analytic code; any other materials used in the review.	N/R

For peer review only - http://bmjopen.bmj.com/site/about/guidelines.xhtml

From: Page MJ, McKenzie JE, Bossuyt PM, Boutron I, Hoffmann TC, Mulrow CD, et al. The PRISMA 2020 statement: an updated guideline for reporting systematic reviews. BMJ 2021;372:n71. doi: 10.1136/bmj.n71

For peer review only

PRISMA 2020 Checklist

http://www.prisma-statement.org/

For peer review only

BMJ Open

Clinical and economic burden of low back pain in low- and middle-income countries: a systematic review

Journal:	*BMJ Open*
Manuscript ID	bmjopen-2022-064119.R1
Article Type:	Original research
Date Submitted by the Author:	19-Dec-2022
Complete List of Authors:	Fatoye, Francis; Manchester Metropolitan University<br>Gebrye, Tadesse; Manchester Metropolitan University<br>Mbada, Chidozie Emmanuel; Obafemi Awolowo University, Department of Medical Rehabilitation<br>Useh, Ushotanefe; North West Univ,
**Primary Subject Heading**:	Global health
Secondary Subject Heading:	Health economics, Health policy
Keywords:	Health economics < HEALTH SERVICES ADMINISTRATION & MANAGEMENT, PAIN MANAGEMENT, Spine < ORTHOPAEDIC & TRAUMA SURGERY, Back pain < ORTHOPAEDIC & TRAUMA SURGERY

SCHOLARONE™
Manuscripts

I, the Submitting Author has the right to grant and does grant on behalf of all authors of the Work (as defined in the below author licence), an exclusive licence and/or a non-exclusive licence for contributions from authors who are: i) UK Crown employees; ii) where BMJ has agreed a CC-BY licence shall apply, and/or iii) in accordance with the terms applicable for US Federal Government officers or employees acting as part of their official duties; on a worldwide, perpetual, irrevocable, royalty-free basis to BMJ Publishing Group Ltd ("BMJ") its licensees and where the relevant Journal is co-owned by BMJ to the co-owners of the Journal, to publish the Work in this journal and any other BMJ products and to exploit all rights, as set out in our licence.

The Submitting Author accepts and understands that any supply made under these terms is made by BMJ to the Submitting Author unless you are acting as an employee on behalf of your employer or a postgraduate student of an affiliated institution which is paying any applicable article publishing charge ("APC") for Open Access articles. Where the Submitting Author wishes to make the Work available on an Open Access basis (and intends to pay the relevant APC), the terms of reuse of such Open Access shall be governed by a Creative Commons licence – details of these licences and which Creative Commons licence will apply to this Work are set out in our licence referred to above.

Other than as permitted in any relevant BMJ Author's Self Archiving Policies, I confirm this Work has not been accepted for publication elsewhere, is not being considered for publication elsewhere and does not duplicate material already published. I confirm all authors consent to publication of this Work and authorise the granting of this licence.

For peer review only

Clinical and Economic Burden of Low Back Pain in Low- and Middle-Income Countries: A Systematic Review

Francis Fatoye[1, 3], Tadesse Gebrye[1], Chidozie Mbada [2], Ushotanefe Useh [3]

[1] Department of Health Professions, Manchester Metropolitan University, Birley Fields Campus, Bonsall Street, Manchester, M156GX.

[2] Department of Medical Rehabilitation, College of Health Sciences, Obafemi Awolowo University, Ile-Ife, Nigeria.

[3] Lifestyle Diseases, Faculty of Health Sciences, North-West University, South Africa

Correspondence author: Professor Francis Fatoye,

Department of Health Professions,

Faculty of health, Psychology, and Social Care,

Manchester Metropolitan University, Brooks Building,

53 Bonsall Street, Manchester UK, M15 6GX.

Phone: 44 (0) 161 247 2963

Email: t.gebrye@mmu.ac.uk

For peer review only - http://bmjopen.bmj.com/site/about/guidelines.xhtml

Abstract

Objectives: Low back pain (LBP) is the leading cause of disability and work absenteeism globally, and it poses significant clinical and economic burden to individuals, health systems and the society. This study aimed to synthesise the clinical and economic burden of LBP in Low- and Middle-Income Countries (LMICs).

Methods: A systematic review following the Preferred Reporting Items for Systematic Reviews and Meta-Analyses guidelines was performed. PubMed, Medline, CINAHL, PsycINFO, AMED, Embase and Scopus databases were systematically searched for studies that examined the clinical and economic burden of LBP in LMICs, published from inception to December 10, 2021. Only studies with clearly stated methodologies and published in English were eligible for review.

Results: Nine studies met the inclusion criteria and were reviewed. Of these, three of them were clinical burden studies. The mean Newcastle–Ottawa quality assessment scale (NOS) score of the included studies was 4, with an average from 3 to 6. The included studies were conducted in Argentina, Brazil, China, Ethiopia, Nigeria, and Republic of Serbia. The rates of hospitalisation due to LBP ranged between 13.4% to 18.7%. Due to variation of methodological approaches, the reported cost estimates were inconsistent across the studies. A total cost of US$2.2 billion per population and US$1226.25 per patient were reported annually due to LBP.

Conclusion: This systematic literature review suggests that LBP is associated with significantly high rates of hospitalisation and costs. As LBP is an important threat to the population, health professionals and policymakers are to put in place appropriate programmes to reduce the clinical and economic burden associated with LBP and improve the health outcomes of individuals with this condition in LMICs.

Keywords: Low back pain, economic, clinical, LMICs

Strength and limitations of the study

- This is the first study that summarises and critically appraises the current evidence base on clinical and economic impact of LBP in LMICs and discusses the potential avenues for future research.
- This review covers a wide variety of the literature and follows the Preferred Reporting Items for Systematic Reviews and Meta-Analyses.
- The included literature were small and failed to fully report their data which impacted the quality ratings and the content of the meta-analyses.

For peer review only - http://bmjopen.bmj.com/site/about/guidelines.xhtml

Introduction

Low-back pain (LBP) is an important health problem and covers radicular, axial lumbosacral, and referred pain [1]. The lifetime, 1-year and point prevalence of LBP worldwide ranged from 11% to 84%, 22% to 65% and 12% to 33%, respectively [2]. The 1-year incidence of people who have a first-ever episode and any episode of LBP ranged 6.5% to 15.4% and 1.5% to 36% [3]. LBP is associated with increased health care costs, length of stay in hospitals, morbidity and mortality in both high- and low-income countries [4].

To reduce the clinical and economic burden associated with LBP, it is important that prevention and management strategies of LBP are implemented. Prevention and management strategies are aimed at controlling pain, maintaining function and prevention of exacerbation of LBP [5]. The American College of Physicians and the American Pain Society recommends that individual with LBP should remain active, and they provide information about effective self-care options, use of medications with proven benefits, interdisciplinary rehabilitation, exercise, acupuncture, massage, spinal manipulation, yoga, cognitive behavioural therapy, or relaxation for the treatment of LBP [6].

The implementation of these treatments remains a challenge for clinical practice and research [7]. Because of this challenge, LBP was listed among the top 10 diseases and injuries that account for the highest number of disability-adjusted life years (DALYs) worldwide [8]. The direct medical and indirect costs of LBP are more than $50 billion per annum and could be as high as $100 billion at the extreme [9]. Evidence suggests that as much as 80% to 90% of the populations' work in low- and middle-income countries (LMICs) entails heavy labour such as carrying heavy loads on the back or head [10]. The risk factors of LBP include low educational status, stress, anxiety, depression, job dissatisfaction, low levels of social support in the workplace and whole-body vibration [3]. The causes and risk factors for LBP in LMIC is substantial [11]. Thus, the likelihood of having LBP is higher in LMICs than high income countries.

Many studies have been published evaluating the clinical and economic burden of LBP in LMICs. However, to date there are no studies that have summarised literature on the clinical and economic burden of LBP in LMICs. Therefore, this systematic review critically evaluated and summarised the results of all available published systematic reviews that have investigated the clinical and economic burden of LBP in LMICs.

Methods

Search protocol and registration

In this study, we used the Preferred Reporting Items for Systematic Reviews and Meta-Analysis guideline. A protocol for this systematic review was prospectively registered on PROSPERO and can be found at https://www.crd.york.ac.uk/prospero/#recordDetails ID = CRD42020196335.

For peer review only - http://bmjopen.bmj.com/site/about/guidelines.xhtml

Search strategy

The systematic review was carried out in the PubMed, Medline, CINAHL, PsycINFO, AMED, Embase and Scopus databases with studies published from inception to December 10, 2021.The following keywords were used in the search: low back pain, hospitalisation, cost of illness, absenteeism, ambulatory care, drug costs, emergency medical services, healthcare costs, nursing services, economics, physicians visit, clinical impact, utilization, burden of illness, cost, nursing cost (Appendix 1). These search terms were combined using conjunctions words 'AND' or 'OR'. Further, a manual search of reference sections of the included studies was also checked for additional studies. The search was performed independently by two authors (TG & FF) to avoid the presence of bias in the selection and exclusion of studies. Any disagreement was resolved by discussion with the third the author (CEM).

Inclusion and exclusion criteria

Included in this review were studies among patients with LBP; original research findings related to costs (direct and indirect) in LMIC as defined by World Health Organization (WHO); the contexts of interest were hospitals, primary healthcare clinics and home settings; observational (cross-sectional or surveys) published in peer-reviewed journals. Review articles, editorials, letters to the editor, news reports, conference abstracts, comments, languages other than English, as well as the results of dissertations were excluded. For ease of interpretation, access and language barrier, reviews that were published or unpublished in another language were excluded.

Study selection and assessment of methodological quality

After selection of the articles by the search strategy in each database, duplicate articles were excluded. Following the removal of duplicates, titles and abstracts were screened by two reviewers (TG & FF) to identify eligible studies. Articles were selected for inclusion based on predefined criteria like relevant patient population (LBP), appropriate study design and outcome measures (patient- and population-level). Studies assessing the efficacy or effectiveness of specific interventions were not included.

Discrepancies for study selection were resolved through discussion with other investigators (CEM & UU). Having retrieved the full text of studies that met the inclusion criteria, they were assessed for methodological quality using the Newcastle–Ottawa quality assessment scale (NOS) for cohort studies [12]. The NOS contains nine items, categorized into three dimensions including selection and comparability. Studies were scored using a scale with a possible maximum of nine points where a score ≥ 6 indicated high-quality studies, a score between 3 - 6 as moderate and a score ≤ 3 as low quality.

Data extraction

Data were extracted by two independent review authors. The following information was extracted for each study: authors, country and year of publication, study objective, data source, inclusion criteria, LBP definition, population characteristics (size, % male, and mean age), hospitalisation, average total annual cost per patient and annual

For peer review only - http://bmjopen.bmj.com/site/about/guidelines.xhtml

population cost. Hospitalization was defined as a primary discharge diagnosis of LBP, it could be obtained from hospital database records of discharge and admission date [13]. Summary table was used to display the extracted data. When there was disagreement, it was addressed through consultation with the third reviewer (CEM).

Data analysis

Given the heterogeneity of included studies in the review, it was not possible to undertake a meta-analysis. Instead, a narrative synthesis of the extracted data was employed to analyse, integrate, and synthesise review findings. Study characteristics were firstly tabulated along with extracted outcomes to support the narrative synthesis. A preliminary synthesis was then undertaken by categorising extracted data clusters by reference/country, data source, study objective, inclusion criteria, LBP definition and clinical and economic burden of LBP. Using the tabulated data, relationships within and between included studies were then examined visually.

If necessary, costs were converted to US$ using Salary converter [14]. Most of costs have been converted to US$ using purchasing power party and then inflation adjustments were calculated using the suggested country specific inflation index from the from the World Bank Website [15]. Conversions were performed in February 2022. Further, hospitalization rates were the number of LBP patients hospitalised in a given period of follow-up. The higher rate of hospitalisation could mean substantial clinical burden of the condition.

Patient and public involvement

Patients and the public were not involved in the design or planning of the study.

Results

This systematic review generated 3,074 records in Scopus (n = 51), PubMed (n = 2,036), Embase (n = 700) and Medline, AMED, CINAHL, PsycINFO (n = 287) (Figure 1). Of these, 278 were duplicates. Following screening by titles and abstracts 2,766 studies were excluded, leaving 30 articles for further full text review. No article was added through manual search of the bibliographies of relevant articles. After reading the full text only 9 met the inclusion criteria and were eligible for analysis. The list of the excluded studies was also reported (Appendix 2). The mean NOS score of the included studies was 4, with an average from 3 to 6 (Table 1). The NOS score showed all the included studies do not show comparability of cohorts on the basis of age, sex the design, and analysis.

....................Figure 1 about here.................

Characteristics of the included studies

The characteristics of the included studies are presented in Table 1. The clinical and economic burden of LBP studies were conducted in Nigeria (n = 3), Ethiopia (n = 1), Brazil (n = 2), China (n = 1), Republic of Serbia (n = 1), and Argentina (n = 1). Of the 9 eligible studies included in our review, 3 studies [16, 17, 18] reported the clinical impact of LBP in LMICs. Whereas the remaining studies reported the economic burden of LBP. The data sources

For peer review only - http://bmjopen.bmj.com/site/about/guidelines.xhtml

of the included studies were interviews, questionnaire, population based cross-sectional study and medical records. Except three studies [16, 19, 20], all the included studies reported LBP Classification or diagnosis.

For peer review only

Table 1: Characteristics of clinical and economic burden studies included in the review

Reference/ Country	Data source	Study objective	Inclusion criteria	Classification or diagnosis LBP	Conflict of interest	Funding source	NOS/9
Beyera et al., [16]/ Ethiopia	Interview	To analyse hospital admission and associated factors	Individuals with LBP of ≥ 18 years of age. Presented to health care facility for LBP in the past 1-year	Not provided	No	Not stated	3
Carregaro et al., [17]/ Brazil	National databases, considering the period 2012–2016.	To estimate the healthcare expenditure and productivity losses related to LBP	≥ 19 years	ICD-10 codes	No	Yes	6
Laffont et al. [18]/ Argentina	The Statistics and Information Department of the Ministry of Health	To analyse the impact of LBP on hospitalisations between 2006 and 2010.	Either adult LBP or lumbosciatica (≥ 18 years)	ICD-10 codes	No	No	6
Odole et al. [19/ Nigeria	Questionnaire	To determine the economic burden of LBP patients in Ibadan	Individuals with LBP receiving physiotherapy in Ibadan	Not provided	Not stated	Not stated	3
do Socorro et al., [20]/Brazil	Questionnaire	To assess resource utilization in the diagnosis, management, and hospitalization of patients	Participants were rheumatologists attending a national rheumatology medical congress.	Not provided	Not stated	No	4
Bello & Muhammad [21] /Nigeria	Questionnaire	To estimate the cost of LBP management	Patients with LBP, allow them to carry out activities for daily living even under difficulties.	ICD-9 codes	No	No	3
Birabi et al. [22]/ Nigeria	Medical records of Hospitals (220 patients)	To report the results of a retrospective study on the direct cost burden of LBP between 2010-2013	Patients whose care commenced from acute care stages, admitted where necessary.	ICD-10 codes	Not stated	Not stated	5
Lin et al. [23]/ Republic of China	Interview	To estimate the 12- month economic cost of LBP among female (1990 to 1991)	Nurses who had experienced LBP within the 12-month period	ICD-10 codes	No	No	3
Radoičlc et al. [24]/ Republic of Serbia	Interview (April 2016 to April 2017).	To estimate the cost of LBP	Diagnosed as M54, which refers to lumbar pain (Dorsalgia)	ICD-10 codes	No	Partially funded	4

Note: ICD = International Classification of Diseases; NOS = Newcastle–Ottawa quality assessment scale

For peer review only - http://bmjopen.bmj.com/site/about/guidelines.xhtml

Definition of LBP

The definition of LBP adopted by the included studies are presented in Table 2. Except two studies [18, 20], all the included studies provided the definition of LBP. The definition adopted by each included study are not the same. For example, two studies [17, 24] used ICD-codes to define LBP. Other study [16] adopted the frequency of LBP report to health institution.

Table 2: Definition of LBP in the included studies

Study	Definition of LBP
Beyera et al., [16]	Individuals who reported at least 1 presentation to any of the health institutions for their LBP in the past 1-year.
Carregaro et al., [17]	ICD-10 codes were used to define the presence of LBP: M40.4 (Other lordosis); M40.5 (Lordosis, unspecified); M51 (Other intervertebral disc disorders); M54.1 (Radiculopathy); M54.3 (Sciatica); M54.4 (Lumbago with sciatica); M54.5 (Low back pain); M54.8 (Other dorsalgia); and M54.9 (Dorsalgia, unspecified).
Laffont et al. [18]	Not provided
Odole et al. [19]	Patients with mechanical LBP who were receiving physiotherapy on an outpatient
do Socorro et al., [20]	Not provided
Bello & Muhammad [21]	Participants diagnosed and managed for LBP in secondary and tertiary hospitals for at least one-year duration. The pain was low in the back and allow them to carry out activities for daily living even under difficulties.
Birabi et al. [22]	Patients whose ages ranged between 26 and 65years and were managed from acute care period for LBP (without discharge against medical advice) as well as were followed up on out-patient basis without default within the study period.
Lin et al. [23]	The following definition of pain were used: minor: pain occurs when carrying 20 kg of weight for 10 minutes or more; 2) moderate: pain occurs when lifting 20 kg of weight; 3) serious: pain occur; when lifting less than 20 kg of weight; 4) severe: pain occurs when doing any task; 5) maximum: pain occurs even while at rest.
Radoičlc et al. [24]	Patients should have been diagnosed as M54, which refers to lumbar pain (Dorsalgia).

For peer review only - http://bmjopen.bmj.com/site/about/guidelines.xhtml

Hospitalisation

A total of 3 studies reported data on rates of hospitalisation for LBP (Table 3). The rates of hospitalisation ranged between 13.4% to 18.7%. The highest rate of hospitalisation (18.7%) for LBP was reported in Argentina [18].

Table 3: Rate of hospitalisation for LBP patients

Study	Number of participants	Year	Hospitalizations for LBP
Beyera et al. [16]	543 (M = 316; F = 205)	Between June and November 2018	14.4%, [95% CI 11.4-17.3] With an average length of stay 7.4 days, 95% CI 6.4-8.8
Carregaro et al. [17]	886,523 diagnostic imaging procedures for LBP	2012 to 2016	13.4% (inpatient admissions)
Laffont et al. [18]	A total of 154,430 discharges in which patients of any age were hospitalised	2006 and 2010	18.7%, With an average length of stay in the hospital of 3.8 days
do Socorro et al. [20]	207 rheumatologists (M = 62%; F = 38%)	N/A	An average of 574 patients with LBP were seen per year.

Note: N/A = Not available; M = Male; F = Female

Economic burden

The economic burden of the included studies is provided in Table 4. A total of 6 studies reported average annual cost per patient [16, 19, 21, 22, 23, 24]. One of the included studies reported annual cost per population [18]. Patients with the age range up to 85 years were included in the included studies. Due to variation in methodological approaches, the reported cost estimates were inconsistent across the studies. However, up to an average of annual costs of US$2.2 billion and US$1226.25 per population and per patient level, respectively were reported. Up to $1.7 billion indirect costs were also reported mainly due to work absenteeism [18].

For peer review only

Table 4: Summary of studies that reported patient and population -level total costs for LBP

Reference	Age group (years)	Average annual cost per patient	Inflated 2022 $US
Carregaro et al. [17]	≥ 19	Total costs = US$ 2.2 billion¶<br>Indirect cost = 1.7 billion ¶ (productivity losses)	Total cost = US$2.5 billion¶
Odole et al. [19]	30 to 80	Total cost = US$1125.27 ± 623.39<br>Direct costs = US$927.20 ± 600.26.<br>Indirect cost = US$198.08 ± 136.15	Total cost = US$5047.5 ± 2796.3<br>Direct costs = US$4158.1 ± 2691);<br>Indirect cost = US$888 ± 610.
do Socorro et al. [20]	N/A	30% of patients with LBP would be absent from their work due to the condition.	N/A
Bello & Muhammad, [21]	30 to 70	Total = US$763.27 ± 543.97;<br>Direct cost = US$647.28 ± 438.99;<br>Indirect cost = US$115.99 ± 104.99	Total = US$ 3029.9 ± 2159.1<br>Direct cost = US$2569.5 ± 1742.6<br>Indirect cost = 460.1 ± 416.5
Birabi et al. [22]	26 and 65	Total cost = US$1226.25 (government hospitals); US$4884.37 (private hospitals)	#US$3192.53 (government hospitals)<br>#US$12716.41(private hospitals)
Lin et al. [23]	Mean age = 32.2 ± 9.2	Total costs = US$994.4 to US$1406.4.	N/A
Radoičlc et al. [24]	Median, 53.59 (28 – 85)	Total costs = €200.40 ± €86.65,<br>Direct costs = €9.39 ± €6.66.<br>Indirect costs = €182.00 ± €78.66	Total cost = €432.86 ± €187.2<br>Total indirect costs = €393.12 ± €169.99

Note: ¶ = per population; N/A = Not available

Discussion

To our knowledge this is the first systematic review to analyse the clinical and economic impact of LBP in LMICs. A total of 9 studies from six LMICs were included. Our review indicates that the clinical and economic burden of LBP is substantial in LMICs. The rate of hospitalisation ranged between 13.4% and 18.7% of patients with LBP. The findings of the review also suggest that LBP is associated with an annual cost of US$2.2 billion per population in LMICs. A clearer understanding of the clinical and economic burden of LBP in LMICs is therefore important for policy makers that they may consider appropriate prevention and management strategies for LBP.

Rate of hospitalisation of female patients with LBP was higher (53.7%) than their male counterpart [20]. The reason for this could be epidemiological where the proportion of LBP in female is in a higher proportion than males [25]. The average length of stay in hospital due to LBP in Argentina and Ethiopia was 3.8 days [20] and 7.4 days [17], respectively. The length of stay in hospital is directly associated with high financial impact of LBP, this is because of its healthcare and social costs [26].

We collected evidence of both direct and indirect economic burden of LBP. The overall burden varied based on characteristics of patient populations, geographic region and the methodologies adopted by the studies, this includes the individual patient or the population level perspective. For example, the societal annual costs amounted to US$ 2.2 billion per population where the productivity losses represented 79% of the costs reported in Brazil [18]. Overall, the considerable clinical and economic burden of LBP in LMICs could be due to its poorly equipped health and social system [27].

For peer review only - http://bmjopen.bmj.com/site/about/guidelines.xhtml

There are certain strength and limitations to this study. The main strength of this review is the comprehensiveness of the search terms, screening of numerous data bases, and assessment of methodological quality of the studies. Only English language studies were included. Therefore, it is possible that relevant literature published in other languages may have been excluded. it was not possible to undertake a meta-analysis for most of the included studies due to the adoption of different time horizons, different clinical outcomes and lack of data regarding mean and SD for costs [28]. Further, the quality of the included studies indicates that there is a need to interpret the clinical and economic burden results with caution.

In conclusion: We found that LBP is associated with significantly high rate of hospitalisation with a clear impact on length of stay in hospital and treatment costs. The findings of our analysis can be used by clinicians, and policymakers to better allocate resources for prevention and management strategies for LBP improve health outcomes and reduce the substantial burden associated with the condition. We also hope our results will be of use to researchers planning to evaluate the cost-effectiveness of various strategies for preventing LBP in LMICs.

Authors contribution: F.F. & T.G. performed electronic searches and assessed title and abstract. C.EM. and U.U. were consulted in case of disagreement. F.F. & T.G. performed the methodological quality assessment and extracted the data. T.G. & C.EM. wrote the original draft preparation. F.F. & U.U. reviewed, revised, and edited this paper. All authors read and approved the final draft of the manuscript**.**

Ethical approval: For this study ethical approval is not required.

Competing interest: The authors have no competing interests to declare.

Funding information: This research did not receive any specific grant from funding agencies in the public, commercial, or not-for-profit sectors.

Informed consent: The participants' written consent was not required in this study because this was a systematic review study.

Data sharing: All data related to this work are available.

For peer review only

References

1. Urits I, Burshtein A, Sharma M, Testa L, Gold PA, Orhurhu V, Viswanath O, Jones MR, Sidransky MA, Spektor B, Kaye AD. Low back pain, a comprehensive review: pathophysiology, diagnosis, and treatment. Current pain and headache reports. 2019 Mar;23(3):1-0.
2. Tay M, Sian SC, Eow CZ, Ho KL, Ong JH, Sirisena D. Ultrasound-Guided Lumbar Spine Injection for Axial and Radicular Pain: A Single Institution Early Experience. Asian Spine Journal. 2021 Apr;15(2):216.
3. Hoy D, Brooks P, Blyth F, Buchbinder R. The epidemiology of low back pain. Best practice & research Clinical rheumatology. 2010 Dec 1;24(6):769-81.
4. Dagenais S, Caro J, Haldeman S. A systematic review of low back pain cost of illness studies in the United States and internationally. The spine journal. 2008 Jan 1;8(1):8-20.
5. Kuritzky L, Samraj GP. Nonsteroidal anti-inflammatory drugs in the treatment of low back pain. Journal of Pain Research. 2012;5:579.
6. Chou R, Qaseem A, Snow V, Casey D, Cross Jr JT, Shekelle P, Owens DK, Clinical Efficacy Assessment Subcommittee of the American College of Physicians and the American College of Physicians/American Pain Society Low Back Pain Guidelines Panel*. Diagnosis and treatment of low back pain: a joint clinical practice guideline from the American College of Physicians and the American Pain Society. Annals of internal medicine. 2007 Oct 2;147(7):478-91.
7. Koes BW, Van Tulder M, Lin CW, Macedo LG, McAuley J, Maher C. An updated overview of clinical guidelines for the management of non-specific low back pain in primary care. European Spine Journal. 2010 Dec;19(12):2075-94.
8. Vos T, Flaxman AD, Naghavi M, Lozano R, Michaud C, Ezzati M, Shibuya K, Salomon JA, Abdalla S, Aboyans V, Abraham J. Years lived with disability (YLDs) for 1160 sequelae of 289 diseases and injuries 1990–2010: a systematic analysis for the Global Burden of Disease Study 2010. The lancet. 2012 Dec 15;380(9859):2163-96.
9. Hoy D, March L, Brooks P, Blyth F, Woolf A, Bain C, Williams G, Smith E, Vos T, Barendregt J, Murray C. The global burden of low back pain: estimates from the Global Burden of Disease 2010 study. Annals of the rheumatic diseases. 2014 Jun 1;73(6):968-74.
10. Louw QA, Morris LD, Grimmer-Somers K. The prevalence of low back pain in Africa: a systematic review. BMC Musculoskeletal disorders. 2007 Dec;8(1):1-4.
11. Stewart Williams J, Ng N, Peltzer K, Yawson A, Biritwum R, Maximova T, Wu F, Arokiasamy P, Kowal P, Chatterji S. Risk factors and disability associated with low back pain in older adults in low-and middle-income countries. Results from the WHO Study on Global AGEing and Adult Health (SAGE). PloS one. 2015 Jun 4;10(6):e0127880.
12. Stang A. Critical evaluation of the Newcastle-Ottawa scale for the assessment of the quality of nonrandomized studies in meta-analyses. European journal of epidemiology. 2010 Sep;25(9):603-5.

For peer review only - http://bmjopen.bmj.com/site/about/guidelines.xhtml

13. Suissa S, Ernst P, Kezouh A. Regular use of inhaled corticosteroids and the long term prevention of hospitalisation for asthma. Thorax. 2002 Oct 1;57(10):880-4.
14. Salary converter. Purchasing power parities. 2017 http:// salaryconverter. nigelb. me/ (accessed February 2022).
15. World Bank. World Bank open data. 2022. Available at: https://data.worldbank.org/indicator/FP.CPI. TOTL. Accessed February 2022.
16. Beyera GK, O'Brien J, Campbell S. Hospital admission and associated factors among individuals presenting to healthcare facilities for low back pain in Ethiopia. International Journal of Rheumatic Diseases. 2020 Jun;23(6):763-71.
17. Carregaro RL, Tottoli CR, Rodrigues DD, Bosmans JE, da Silva EN, van Tulder M. Low back pain should be considered a health and research priority in Brazil: Lost productivity and healthcare costs between 2012 to 2016. PloS one. 2020 Apr 1;15(4):e0230902.
18. Laffont M, Sequeira G, Kerzberg EM, Marconi E, Guevel C, de las Mercedes Fernández M. The non-silent epidemic: low back pain as a primary cause of hospitalisation. Rheumatology international. 2016 May;36(5):673-7.
19. Odole AC, Akinpelu AA, Adekanla BA, Obisanya OB. Economic burden of low back pain on patients seen at the outpatient Physiotherapy clinics of secondary and tertiary health institutions in Ibadan. Journal of the Nigeria Society of Physiotherapy. 2011 Dec 28;18(1):43-8.
20. do Socorro Margarido M, Kowalski SC, Natour J, Ferraz MB. Acute low back pain: diagnostic and therapeutic practices reported by Brazilian rheumatologists. Spine. 2005 Mar 1;30(5):567-71.
21. Bello B, Muhammad IT. Estimation of economic cost of low back pain management in Kano, Nigeria. Adv Life Sci Med. 2017;3:01-8.
22. Birabi, BN, Oke, KI, Dienye, PO, & Okafor, UAC. Cost Burden of Low Back Pain Amon Adults in Southern Nigeria. International Journal of Medical and Applied Science, 2014 June 4; 3 (3): 24-29.
23. Lin MR, Tsauo JY, Wang JD. Determinants of economic cost related to low back pain among nurses at a University Hospital. International Journal of Occupational and Environmental Health. 1996 Oct 1;2(4):257-63.
24. Radoičlc M, Božovic B, Ilić K, Jankovic S, AnĐelković J, Kostic M. Pharmacoeconomic aspects of low back pain treatment: Cost of illness study in the republic of Serbia. Acta Medica Portuguesa. 2019 Apr 1.
25. Hoy D, Bain C, Williams G, March L, Brooks P, Blyth F, Woolf A, Vos T, Buchbinder R. A systematic review of the global prevalence of low back pain. Arthritis & Rheumatism. 2012 Jun;64(6):2028-37.
26. Anema JR, Schellart AJ, Cassidy JD, Loisel P, Veerman TJ, Van der Beek AJ. Can cross country differences in return-to-work after chronic occupational back pain be explained? An exploratory analysis on disability policies in a six country cohort study. Journal of occupational rehabilitation. 2009 Dec;19(4):419-26.

For peer review only - http://bmjopen.bmj.com/site/about/guidelines.xhtml

27. Hartvigsen J, Hancock MJ, Kongsted A, Louw Q, Ferreira ML, Genevay S, Hoy D, Karppinen J, Pransky G, Sieper J, Smeets RJ. What low back pain is and why we need to pay attention. The Lancet. 2018 Jun 9;391(10137):2356-67.
28. Higgins MD, Green RJ, Leeson MS. Optical wireless for intravehicle communications: A channel viability analysis. IEEE Transactions on Vehicular Technology. 2011 Nov 18;61(1):123-9.

For peer review only

For peer review only - http://bmjopen.bmj.com/site/about/guidelines.xhtml

Figure 1. Flow diagram of publications included and excluded in the review

For peer review only

For peer review only - http://bmjopen.bmj.com/site/about/guidelines.xhtml

Figure

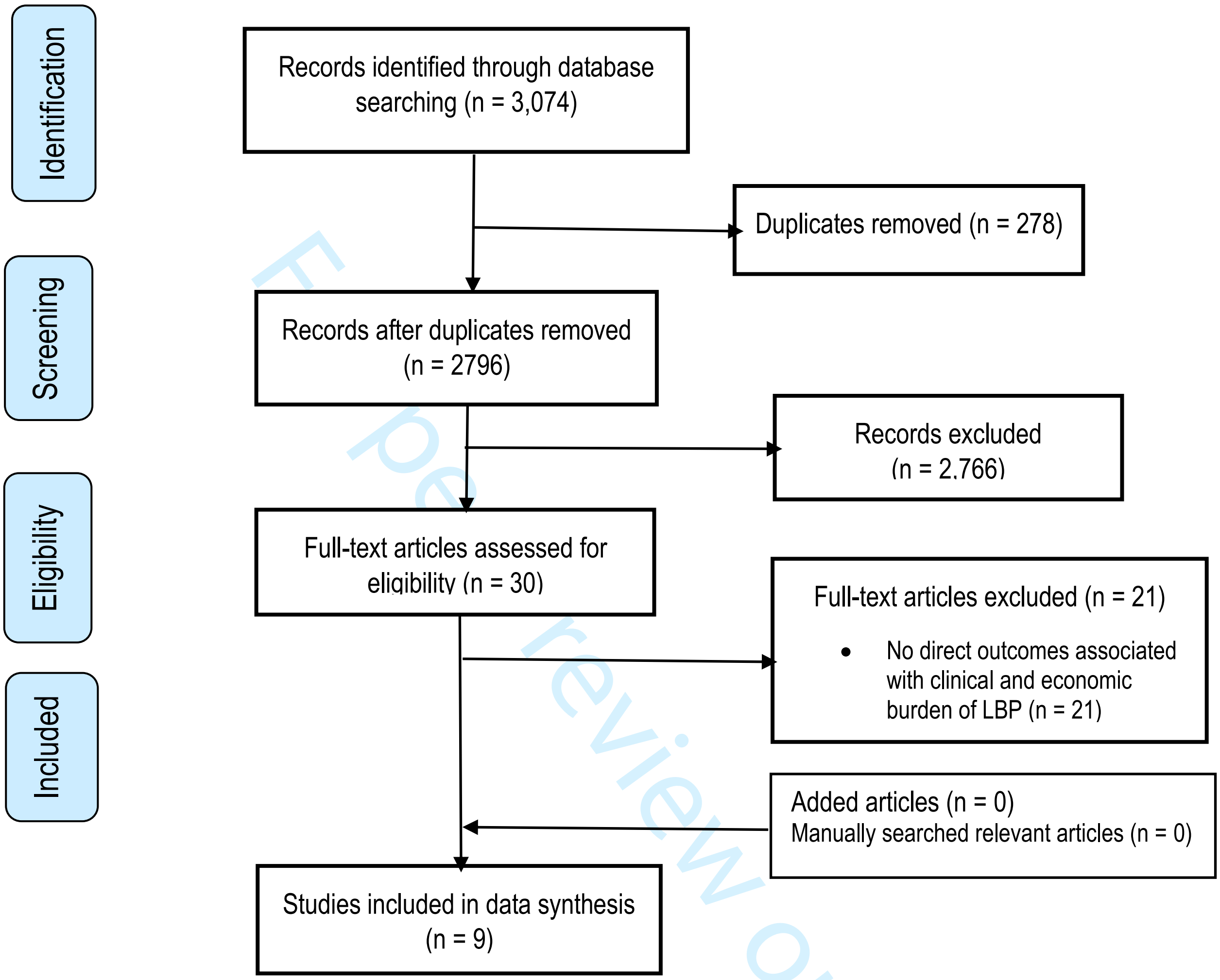

Figure 1. Flow diagram of publications included and excluded in the review

For peer review only

Appendix 1: Clinical and economic burden search strategy

Appendix 1a: Medline, CINAHL, Embase and AMED (inception to 10th December/2021

Search ID	Search Terms
S23	S21 AND S22
S22	S1 OR S2 OR S3 OR S4 OR S5 OR S6 OR S7 OR S8 OR S9 OR S10 OR S11 OR S12 OR S13 OR S14 OR S15 OR S16 OR S17 OR S18 OR S19 OR S20
S21	AB low back pain
S20	AB hospitalization
S19	AB cost of illness
S18	AB absenteeism
S17	AB ambulatory care
S16	AB drug cost
S15	AB emergency medical services
S14	AB healthcare services
S13	AB nursing services
S12	AB economics
S11	AB physician
S10	AB burden
S9	AB clinical impact
S8	AB utilization
S7	AB burden of illness
S6	AB cost
S5	AB nursing costs
S4	AB physician cost
S3	AB physician visits
S2	AB emergency department visits
S1	AB years lived with disability

For peer review only

Appendix 1b: Scopus (inception to 10th December/2021

Search ID	Search Terms
1	TITLE-ABS-KEY (low AND back AND pain)
2	TITLE-ABS-KEY (hospitalisation)
3	TITLE-ABS-KEY (cost AND of AND illness)
4	TITLE-ABS-KEY (absenteeism)
5	TITLE-ABS-KEY (ambulatory AND care)
6	TITLE-ABS-KEY (drug AND costs)
7	TITLE-ABS-KEY (emergency AND medical AND services)
8	TITLE-ABS-KEY (healthcare AND costs)
9	TITLE-ABS-KEY (nursing AND services)
10	TITLE-ABS-KEY (economics)
11	TITLE-ABS-KEY (physicians)
12	TITLE-ABS-KEY (burden)
13	TITLE-ABS-KEY (clinical AND impact)
14	TITLE-ABS-KEY (utilization)
15	TITLE-ABS-KEY (burden AND of AND illness)
16	TITLE-ABS-KEY (cost)
17	TITLE-ABS-KEY (nursing AND cost)
18	TITLE-ABS-KEY (physician AND cost)
19	TITLE-ABS-KEY (physician AND visit)
20	(TITLE-ABS-KEY (hospitalisation)) OR (TITLE-ABS-KEY (cost AND of AND illness)) OR (TITLE-ABS-KEY (absenteeism)) OR (TITLE-ABS-KEY (ambulatory AND care)) OR (TITLE-ABS-KEY (drug AND costs)) OR (TITLE-ABS-KEY (emergency AND medical AND services)) OR (TITLE-ABS-KEY (healthcare AND costs)) OR (TITLE-ABS-KEY (nursing AND services)) OR (TITLE-ABS-KEY (economics)) OR (TITLE-ABS-KEY (physicians)) OR (TITLE-ABS-KEY (burden)) OR (TITLE-ABS-KEY (clinical AND impact)) OR (TITLE-ABS-KEY (utilization)) OR (TITLE-ABS-KEY (burden AND of AND illness)) OR (TITLE-ABS-KEY (cost)) OR (TITLE-ABS-KEY (nursing AND cost)) OR (TITLE-ABS-KEY (physician AND cost)) OR (TITLE-ABS-KEY (physician AND visit))
21	(TITLE-ABS-KEY (low AND back AND pain)) AND ((TITLE-ABS-KEY (hospitalisation)) OR (TITLE-ABS-KEY (cost AND of AND illness)) OR (TITLE-ABS-KEY (absenteeism)) OR (TITLE-ABS-KEY (ambulatory AND care)) OR (TITLE-ABS-KEY (drug AND costs)) OR (TITLE-ABS-KEY (emergency AND medical AND services)) OR (TITLE-ABS-KEY (healthcare AND costs)) OR (TITLE-ABS-KEY (nursing AND services)) OR (TITLE-ABS-KEY (economics)) OR (TITLE-ABS-KEY (physicians)) OR (TITLE-ABS-KEY (burden)) OR (TITLE-ABS-KEY (clinical AND impact)) OR (TITLE-ABS-KEY (utilization)) OR (TITLE-ABS-KEY (burden AND of AND illness)) OR (TITLE-ABS-KEY (cost)) OR (TITLE-ABS-KEY (nursing AND cost)) OR (TITLE-ABS-KEY (physician AND cost)) OR (TITLE-ABS-KEY (physician AND visit)))

Appendix 1c: PubMed (inception to 10th December/2021

Query	Search Details
(((((((((((((((((Hospitalisation[Title/Abstract] AND ((humans[Filter]) AND (english[Filter])))) OR (cost of illness[Title/Abstract] AND ((humans[Filter]) AND (english[Filter]))))) OR (Absenteeism[Title/Abstract] AND ((humans[Filter]) AND (english[Filter]))))) OR (Ambulatory care[Title/Abstract] AND ((humans[Filter]) AND (english[Filter]))))) OR (Drug costs[Title/Abstract] AND ((humans[Filter]) AND (english[Filter]))))) OR (Emergency medical services[Title/Abstract] AND ((humans[Filter]) AND (english[Filter]))))) OR (healthcare costs[Title/Abstract] AND ((humans[Filter]) AND (english[Filter]))))) OR (nursing services[Title/Abstract] AND ((humans[Filter]) AND (english[Filter]))))) OR (economics[Title/Abstract] AND ((humans[Filter]) AND (english[Filter]))))) OR (physicians[Title/Abstract] AND ((humans[Filter]) AND (english[Filter]))))) OR (burden[Title/Abstract] AND ((humans[Filter]) AND (english[Filter]))))) OR (clinical impact[Title/Abstract] AND ((humans[Filter]) AND (english[Filter]))))) OR (Utilization[Title/Abstract] AND ((humans[Filter]) AND (english[Filter]))))) OR (burden of illness[Title/Abstract] AND ((humans[Filter]) AND (english[Filter]))))) OR (cost[Title/Abstract] AND ((humans[Filter]) AND (english[Filter]))))) OR (nursing cost[Title/Abstract] AND ((humans[Filter]) AND (english[Filter]))))) OR (physician visit[Title/Abstract] AND ((humans[Filter]) AND (english[Filter]))))) AND (low back pain[Title/Abstract] AND ((humans[Filter]) AND (english[Filter])))	((("Hospitalisation"[Title/Abstract] AND ("humans"[MeSH Terms] AND "english"[Language])) OR ("cost of illness"[Title/Abstract] AND ("humans"[MeSH Terms] AND "english"[Language])) OR ("Absenteeism"[Title/Abstract] AND ("humans"[MeSH Terms] AND "english"[Language])) OR ("ambulatory care"[Title/Abstract] AND ("humans"[MeSH Terms] AND "english"[Language])) OR ("drug costs"[Title/Abstract] AND ("humans"[MeSH Terms] AND "english"[Language])) OR ("emergency medical services"[Title/Abstract] AND ("humans"[MeSH Terms] AND "english"[Language])) OR ("healthcare costs"[Title/Abstract] AND ("humans"[MeSH Terms] AND "english"[Language])) OR ("nursing services"[Title/Abstract] AND ("humans"[MeSH Terms] AND "english"[Language])) OR ("economics"[Title/Abstract] AND ("humans"[MeSH Terms] AND "english"[Language])) OR ("physicians"[Title/Abstract] AND ("humans"[MeSH Terms] AND "english"[Language])) OR ("burden"[Title/Abstract] AND ("humans"[MeSH Terms] AND "english"[Language])) OR ("clinical impact"[Title/Abstract] AND ("humans"[MeSH Terms] AND "english"[Language])) OR ("Utilization"[Title/Abstract] AND ("humans"[MeSH Terms] AND "english"[Language])) OR ("burden of illness"[Title/Abstract] AND ("humans"[MeSH Terms] AND "english"[Language])) OR ("cost"[Title/Abstract] AND ("humans"[MeSH Terms] AND "english"[Language])) OR ("nursing cost"[Title/Abstract] AND ("humans"[MeSH Terms] AND "english"[Language])) OR ("physician visit"[Title/Abstract] AND ("humans"[MeSH Terms] AND "english"[Language]))) AND ("low back pain"[Title/Abstract] AND ("humans"[MeSH Terms] AND "english"[Language]))) AND ((humans[Filter]) AND (english[Filter]))
low back pain[Title/Abstract]	("low back pain"[Title/Abstract]) AND ((humans[Filter]) AND (english[Filter]))
physician visit[Title/Abstract]	("physician visit"[Title/Abstract]) AND ((humans[Filter]) AND (english[Filter]))
nursing cost[Title/Abstract]	("nursing cost"[Title/Abstract]) AND ((humans[Filter]) AND (english[Filter]))
cost[Title/Abstract]	("cost"[Title/Abstract]) AND ((humans[Filter]) AND (english[Filter]))
burden of illness[Title/Abstract]	("burden of illness"[Title/Abstract]) AND ((humans[Filter]) AND (english[Filter]))
utilization[Title/Abstract]	("Utilization"[Title/Abstract]) AND ((humans[Filter]) AND (english[Filter]))
clinical impact[Title/Abstract]	("clinical impact"[Title/Abstract]) AND ((humans[Filter]) AND (english[Filter]))
burden[Title/Abstract]	("burden"[Title/Abstract]) AND ((humans[Filter]) AND (english[Filter]))
physicians[Title/Abstract]	("physicians"[Title/Abstract]) AND ((humans[Filter]) AND (english[Filter]))
economics[Title/Abstract]	("economics"[Title/Abstract]) AND ((humans[Filter]) AND (english[Filter]))
nursing services[Title/Abstract]	("nursing services"[Title/Abstract]) AND ((humans[Filter]) AND (english[Filter]))
healthcare costs[Title/Abstract]	("healthcare costs"[Title/Abstract]) AND ((humans[Filter]) AND (english[Filter]))
emergency medical services[Title/Abstract]	("emergency medical services"[Title/Abstract]) AND ((humans[Filter]) AND (english[Filter]))
Drug costs[Title/Abstract]	("drug costs"[Title/Abstract]) AND ((humans[Filter]) AND (english[Filter]))
Ambulatory care[Title/Abstract]	("ambulatory care"[Title/Abstract]) AND ((humans[Filter]) AND (english[Filter]))
Absenteeism[Title/Abstract]	("Absenteeism"[Title/Abstract]) AND ((humans[Filter]) AND (english[Filter]))
cost of illness[Title/Abstract]	("cost of illness"[Title/Abstract]) AND ((humans[Filter]) AND (english[Filter]))
hospitalisation[Title/Abstract]	("Hospitalisation"[Title/Abstract]) AND ((humans[Filter]) AND (english[Filter]))

For peer review only - http://bmjopen.bmj.com/site/about/guidelines.xhtml

Appendix 2: List of excluded studies

Study	Country	Design	Aim	Justification for exclusion
Safiri et al., 2021	Global	Model	To determine the burden of other musculoskeletal disorders between 1990 and 2017 at the global, regional and national level across 195 countries and territories	Estimate not for low back pain
Barreto & Sá, 2019	Brazil	Questionnaire	To estimate indirect cost related to chronic pain from an employer's perspective	Estimate not for low back pain
Du et al., 2019	China	CR	To systematically explore the correlates of emotional distress in patients with chronic low back pain	Not clinical or economic burden study.
Driscoll et al., 2014	Global	Servery	To quantify the burden arising from low back pain (LBP) due to occupational exposure to ergonomic risk factors.	Not clinical or economic burden study.
Hoy et al., 2014	Global	Survey	To estimate the global burden of LBP.	Not related to the research topic
Smith et al., 2014	Global	Systematic reviews	To estimate disability from the remainder of musculoskeletal (MSK) disorders	Not clinical or economic burden study
Vos et al., 2012	Worldwide	Model	To describe the approach to undertaking past burden of disease assessments with the available evidence.	Not related
Moradi-Lakeh et al., 2017	EMR	Systematic analysis	To report the burden of musculoskeletal disorders in the Eastern Mediterranean Region.	Not clinical or economic burden study
de David et al., 2020	Brazil	Model	To describe the current status and trends of the burden due to LBP in Brazil	Prevalence and incidence
Amaefule et al., 2021	Nigeria	CR	To estimate the burden of chronic musculoskeletal Disorders amongst patients attending orthopaedic outpatients' clinic	Ful article not available
Aminde et al., 2020	Cameroon	CR	To evaluate health-related quality of life (HRQoL) and its determinants in chronic low back pain patients in Cameroon.	Not clinical or economic burden study
Dagenais et al., 2008	USA	CR	To conduct a systematic review of LBP cost of illness studies	A systematic review
Wu et al., 2019	China	CR	To quantify the prevalence and years lived with disability (YLDs) caused by LBP in China	Not clinical or economic burden study
Dienye et al., 2016	Nigeria	CR	To determine the prevalence of LBP	Prevalence and incidence
Galukande et al., 2006	Uganda	CR	To assess and document the disability associated with low back pain in terms of sick leave	Not clinical or economic burden study
Faezi et al., 2020	Iran	Interviews	To evaluate health-care access and utilization among patients with LBP in Iran	Not clinical or economic burden study
Igwesi-Chidobe et al., 2019	Nigeria	Questionnaire	To assess self-reported disability in a low-literate population with chronic LBP	Not clinical or economic burden study
Michalik et al., 2015	Poland	CR	To present the epidemiology data on back pain in Poland	Prevalence and incidence
Carregaro et al., 2019	Brazil	Prevalence-based	To estimate the direct healthcare costs of spinal disorders in Brazil over 2016.	Not low back pain
Kisa et al., 2020	Global	Systematic analysis	To provides an overview of the influence of occupational risk factors on the global burden of disease	Not low back pain
Maetzel & Li, 2002	N/A	Systematic review	To gain a better understanding of the societal costs of LBP	A review of studies

EMR = Eastern Mediterranean Region; CR = Cross sectional

For peer review only

PRISMA 2020 Checklist

Section and Topic	Item #	Checklist item	Location where item is reported
**TITLE**			
Title	1	Identify the report as a systematic review.	1
**ABSTRACT**			
Abstract	2	See the PRISMA 2020 for Abstracts checklist.	2
**INTRODUCTION**			
Rationale	3	Describe the rationale for the review in the context of existing knowledge.	3
Objectives	4	Provide an explicit statement of the objective(s) or question(s) the review addresses.	3
**METHODS**			
Eligibility criteria	5	Specify the inclusion and exclusion criteria for the review and how studies were grouped for the syntheses.	4
Information sources	6	Specify all databases, registers, websites, organisations, reference lists and other sources searched or consulted to identify studies. Specify the date when each source was last searched or consulted.	4
Search strategy	7	Present the full search strategies for all databases, registers and websites, including any filters and limits used.	4
Selection process	8	Specify the methods used to decide whether a study met the inclusion criteria of the review, including how many reviewers screened each record and each report retrieved, whether they worked independently, and if applicable, details of automation tools used in the process.	4
Data collection process	9	Specify the methods used to collect data from reports, including how many reviewers collected data from each report, whether they worked independently, any processes for obtaining or confirming data from study investigators, and if applicable, details of automation tools used in the process.	4
Data items	10a	List and define all outcomes for which data were sought. Specify whether all results that were compatible with each outcome domain in each study were sought (e.g. for all measures, time points, analyses), and if not, the methods used to decide which results to collect.	5
	10b	List and define all other variables for which data were sought (e.g. participant and intervention characteristics, funding sources). Describe any assumptions made about any missing or unclear information.	5
Study risk of bias assessment	11	Specify the methods used to assess risk of bias in the included studies, including details of the tool(s) used, how many reviewers assessed each study and whether they worked independently, and if applicable, details of automation tools used in the process.	4
Effect measures	12	Specify for each outcome the effect measure(s) (e.g. risk ratio, mean difference) used in the synthesis or presentation of results.	N/R
Synthesis methods	13a	Describe the processes used to decide which studies were eligible for each synthesis (e.g. tabulating the study intervention characteristics and comparing against the planned groups for each synthesis (item #5)).	5
	13b	Describe any methods required to prepare the data for presentation or synthesis, such as handling of missing summary statistics, or data conversions.	5
	13c	Describe any methods used to tabulate or visually display results of individual studies and syntheses.	5
	13d	Describe any methods used to synthesize results and provide a rationale for the choice(s). If meta-analysis was performed, describe the model(s), method(s) to identify the presence and extent of statistical heterogeneity, and software package(s) used.	N/R
	13e	Describe any methods used to explore possible causes of heterogeneity among study results (e.g. subgroup analysis, meta-regression).	N/R
	13f	Describe any sensitivity analyses conducted to assess robustness of the synthesized results.	N/R
Reporting bias assessment	14	Describe any methods used to assess risk of bias due to missing results in a synthesis (arising from reporting biases).	5
Certainty	15	Describe any methods used to assess certainty (or confidence) in the body of evidence for an outcome.	N/R

For peer review only

PRISMA 2020 Checklist

Section and Topic	Item #	Checklist item	Location where item is reported
assessment			
**RESULTS**			
Study selection	16a	Describe the results of the search and selection process, from the number of records identified in the search to the number of studies included in the review, ideally using a flow diagram.	5
	16b	Cite studies that might appear to meet the inclusion criteria, but which were excluded, and explain why they were excluded.	5
Study characteristics	17	Cite each included study and present its characteristics.	6
Risk of bias in studies	18	Present assessments of risk of bias for each included study.	6
Results of individual studies	19	For all outcomes, present, for each study: (a) summary statistics for each group (where appropriate) and (b) an effect estimate and its precision (e.g. confidence/credible interval), ideally using structured tables or plots.	6
Results of syntheses	20a	For each synthesis, briefly summarise the characteristics and risk of bias among contributing studies.	6
	20b	Present results of all statistical syntheses conducted. If meta-analysis was done, present for each the summary estimate and its precision (e.g. confidence/credible interval) and measures of statistical heterogeneity. If comparing groups, describe the direction of the effect.	N/R
	20c	Present results of all investigations of possible causes of heterogeneity among study results.	N/R
	20d	Present results of all sensitivity analyses conducted to assess the robustness of the synthesized results.	N/R
Reporting biases	21	Present assessments of risk of bias due to missing results (arising from reporting biases) for each synthesis assessed.	N/R
Certainty of evidence	22	Present assessments of certainty (or confidence) in the body of evidence for each outcome assessed.	N/R
**DISCUSSION**			
Discussion	23a	Provide a general interpretation of the results in the context of other evidence.	8
	23b	Discuss any limitations of the evidence included in the review.	8
	23c	Discuss any limitations of the review processes used.	8
	23d	Discuss implications of the results for practice, policy, and future research.	8
**OTHER INFORMATION**			
Registration and protocol	24a	Provide registration information for the review, including register name and registration number, or state that the review was not registered.	3
	24b	Indicate where the review protocol can be accessed, or state that a protocol was not prepared.	3
	24c	Describe and explain any amendments to information provided at registration or in the protocol.	N/R
Support	25	Describe sources of financial or non-financial support for the review, and the role of the funders or sponsors in the review.	9
Competing interests	26	Declare any competing interests of review authors.	9
Availability of data, code and other materials	27	Report which of the following are publicly available and where they can be found: template data collection forms; data extracted from included studies; data used for all analyses; analytic code; any other materials used in the review.	N/R

For peer review only - http://bmjopen.bmj.com/site/about/guidelines.xhtml

From: Page MJ, McKenzie JE, Bossuyt PM, Boutron I, Hoffmann TC, Mulrow CD, et al. The PRISMA 2020 statement: an updated guideline for reporting systematic reviews. BMJ 2021;372:n71. doi: 10.1136/bmj.n71

PRISMA 2020 Checklist

http://www.prisma-statement.org/

For peer review only

Clinical and economic burden of low back pain in low- and middle-income countries: a systematic review

Journal:	*BMJ Open*
Manuscript ID	bmjopen-2022-064119.R2
Article Type:	Original research
Date Submitted by the Author:	31-Jan-2023
Complete List of Authors:	Fatoye, Francis; Manchester Metropolitan University<br>Gebrye, Tadesse; Manchester Metropolitan University<br>Mbada, Chidozie Emmanuel; Obafemi Awolowo University, Department of Medical Rehabilitation<br>Useh, Ushotanefe; North West Univ,
**Primary Subject Heading**:	Global health
Secondary Subject Heading:	Health economics, Health policy
Keywords:	Health economics < HEALTH SERVICES ADMINISTRATION & MANAGEMENT, PAIN MANAGEMENT, Spine < ORTHOPAEDIC & TRAUMA SURGERY, Back pain < ORTHOPAEDIC & TRAUMA SURGERY

I, the Submitting Author has the right to grant and does grant on behalf of all authors of the Work (as defined in the below author licence), an exclusive licence and/or a non-exclusive licence for contributions from authors who are: i) UK Crown employees; ii) where BMJ has agreed a CC-BY licence shall apply, and/or iii) in accordance with the terms applicable for US Federal Government officers or employees acting as part of their official duties; on a worldwide, perpetual, irrevocable, royalty-free basis to BMJ Publishing Group Ltd ("BMJ") its licensees and where the relevant Journal is co-owned by BMJ to the co-owners of the Journal, to publish the Work in this journal and any other BMJ products and to exploit all rights, as set out in our licence.

The Submitting Author accepts and understands that any supply made under these terms is made by BMJ to the Submitting Author unless you are acting as an employee on behalf of your employer or a postgraduate student of an affiliated institution which is paying any applicable article publishing charge ("APC") for Open Access articles. Where the Submitting Author wishes to make the Work available on an Open Access basis (and intends to pay the relevant APC), the terms of reuse of such Open Access shall be governed by a Creative Commons licence – details of these licences and which Creative Commons licence will apply to this Work are set out in our licence referred to above.

Other than as permitted in any relevant BMJ Author's Self Archiving Policies, I confirm this Work has not been accepted for publication elsewhere, is not being considered for publication elsewhere and does not duplicate material already published. I confirm all authors consent to publication of this Work and authorise the granting of this licence.

For peer review only

Clinical and Economic Burden of Low Back Pain in Low- and Middle-Income Countries: A Systematic Review

Francis Fatoye[1, 2], Tadesse Gebrye[1], Chidozie Mbada [1], Ushotanefe Useh [2]

[1] Department of Health Professions, Manchester Metropolitan University, Birley Fields Campus, Bonsall Street, Manchester, M156GX.

[2] Lifestyle Diseases, Faculty of Health Sciences, North-West University, South Africa

Correspondence author: Professor Francis Fatoye,

Department of Health Professions,

Faculty of health, Psychology, and Social Care,

Manchester Metropolitan University, Brooks Building,

53 Bonsall Street, Manchester UK, M15 6GX.

Phone: 44 (0) 161 247 2963

Email: t.gebrye@mmu.ac.uk

For peer review only

For peer review only - http://bmjopen.bmj.com/site/about/guidelines.xhtml

Abstract

Objectives: Low back pain (LBP) is the leading cause of disability and work absenteeism globally, and it poses significant clinical and economic burden to individuals, health systems and the society. This study aimed to synthesise the clinical and economic burden of LBP in Low- and Middle-Income Countries (LMICs).

Methods: A systematic review following the Preferred Reporting Items for Systematic Reviews and Meta-Analyses guidelines was performed. PubMed, Medline, CINAHL, PsycINFO, AMED, Embase and Scopus databases were systematically searched for studies that examined the clinical and economic burden of LBP in LMICs, published from inception to December 10, 2021. Only studies with clearly stated methodologies and published in English were eligible for review.

Results: Nine studies met the inclusion criteria and were reviewed. Of these, three of them were clinical burden studies. The mean Newcastle–Ottawa quality assessment scale (NOS) score of the included studies was 4, with an average from 3 to 6. The included studies were conducted in Argentina, Brazil, China, Ethiopia, Nigeria, and Republic of Serbia. The rates of hospitalisation due to LBP ranged between 13.4% to 18.7%. Due to variation of methodological approaches, the reported cost estimates were inconsistent across the studies. A total cost of US$2.2 billion per population and US$1226.25 per patient were reported annually due to LBP.

Conclusion: This systematic literature review suggests that LBP is associated with significantly high rates of hospitalisation and costs. As LBP is an important threat to the population, health professionals and policymakers are to put in place appropriate programmes to reduce the clinical and economic burden associated with LBP and improve the health outcomes of individuals with this condition in LMICs.

Keywords: Low back pain, economic, clinical, LMICs

Strength and limitations of the study

- This is the first study that summarises and critically appraises the current evidence base on clinical and economic impact of LBP in LMICs and discusses the potential avenues for future research.
- This review covers a wide variety of the literature and follows the Preferred Reporting Items for Systematic Reviews and Meta-Analyses.
- The included literature were small and failed to fully report their data which impacted the quality ratings and the content of the meta-analyses.

For peer review only - http://bmjopen.bmj.com/site/about/guidelines.xhtml

Introduction

Low-back pain (LBP) is an important health problem and covers radicular, axial lumbosacral, and referred pain [1]. The lifetime, 1-year and point prevalence of LBP worldwide ranged from 11% to 84%, 22% to 65% and 12% to 33%, respectively [2]. The 1-year incidence of people who have a first-ever episode and any episode of LBP ranged 6.5% to 15.4% and 1.5% to 36% [3]. LBP is associated with increased health care costs, length of stay in hospitals, morbidity and mortality in both high- and low-income countries [4].

To reduce the clinical and economic burden associated with LBP, it is important that prevention and management strategies of LBP are implemented. Prevention and management strategies are aimed at controlling pain, maintaining function and prevention of exacerbation of LBP [5]. The American College of Physicians and the American Pain Society recommends that individual with LBP should remain active, and they provide information about effective self-care options, use of medications with proven benefits, interdisciplinary rehabilitation, exercise, acupuncture, massage, spinal manipulation, yoga, cognitive behavioural therapy, or relaxation for the treatment of LBP [6].

The implementation of these treatments remains a challenge for clinical practice and research [7]. Because of this challenge, LBP was listed among the top 10 diseases and injuries that account for the highest number of disability-adjusted life years (DALYs) worldwide [8]. The direct medical and indirect costs of LBP are more than $50 billion per annum and could be as high as $100 billion at the extreme [9]. Evidence suggests that as much as 80% to 90% of the populations' work in low- and middle-income countries (LMICs) entails heavy labour such as carrying heavy loads on the back or head [10]. The risk factors of LBP include low educational status, stress, anxiety, depression, job dissatisfaction, low levels of social support in the workplace and whole-body vibration [3]. The causes and risk factors for LBP in LMIC is substantial [11]. Thus, the likelihood of having LBP is higher in LMICs than high income countries.

Many studies have been published evaluating the clinical and economic burden of LBP in LMICs. However, to date there are no studies that have summarised literature on the clinical and economic burden of LBP in LMICs. Therefore, this systematic review critically evaluated and summarised the results of all available published systematic reviews that have investigated the clinical and economic burden of LBP in LMICs.

Methods

Search protocol and registration

In this study, we used the Preferred Reporting Items for Systematic Reviews and Meta-Analysis guideline. A protocol for this systematic review was prospectively registered on PROSPERO and can be found at https://www.crd.york.ac.uk/prospero/#recordDetails ID = CRD42020196335.

For peer review only - http://bmjopen.bmj.com/site/about/guidelines.xhtml

Search strategy

The systematic review was carried out in the PubMed, Medline, CINAHL, PsycINFO, AMED, Embase and Scopus databases with studies published from inception to December 10, 2021.The following keywords were used in the search: low back pain, hospitalisation, cost of illness, absenteeism, ambulatory care, drug costs, emergency medical services, healthcare costs, nursing services, economics, physicians visit, clinical impact, utilization, burden of illness, cost, nursing cost (Appendix 1). These search terms were combined using conjunctions words 'AND' or 'OR'. Further, a manual search of reference sections of the included studies was also checked for additional studies. The search was performed independently by two authors (TG & FF) to avoid the presence of bias in the selection and exclusion of studies. Any disagreement was resolved by discussion with the third the author (CEM).

Inclusion and exclusion criteria

Included in this review were studies among patients with LBP; original research findings related to costs (direct and indirect) in LMIC as defined by World Health Organization (WHO); the contexts of interest were hospitals, primary healthcare clinics and home settings; observational (cross-sectional or surveys) published in peer-reviewed journals. Review articles, editorials, letters to the editor, news reports, conference abstracts, comments, languages other than English, as well as the results of dissertations were excluded. For ease of interpretation, access and language barrier, reviews that were published or unpublished in another language were excluded.

Study selection and assessment of methodological quality

After selection of the articles by the search strategy in each database, duplicate articles were excluded. Following the removal of duplicates, titles and abstracts were screened by two reviewers (TG & FF) to identify eligible studies. Articles were selected for inclusion based on predefined criteria like relevant patient population (LBP), appropriate study design and outcome measures (patient- and population-level). Studies assessing the efficacy or effectiveness of specific interventions were not included.

Discrepancies for study selection were resolved through discussion with other investigators (CEM & UU). Having retrieved the full text of studies that met the inclusion criteria, they were assessed for methodological quality using the Newcastle–Ottawa quality assessment scale (NOS) for cohort studies [12]. The NOS contains nine items, categorized into three dimensions including selection and comparability. Studies were scored using a scale with a possible maximum of nine points where a score ≥ 6 indicated high-quality studies, a score between 3 - 6 as moderate and a score ≤ 3 as low quality.

Data extraction

Data were extracted by two independent review authors. The following information was extracted for each study: authors, country and year of publication, study objective, data source, inclusion criteria, LBP definition, population characteristics (size, % male, and mean age), hospitalisation, average total annual cost per patient and annual population cost. Hospitalization was defined as a primary discharge diagnosis of LBP, it could be obtained from

For peer review only - http://bmjopen.bmj.com/site/about/guidelines.xhtml

hospital database records of discharge and admission date [13]. Summary table was used to display the extracted data. When there was disagreement, it was addressed through consultation with the third reviewer (CEM & UU).

Data analysis

Given the heterogeneity of included studies in the review, it was not possible to undertake a meta-analysis. Instead, a narrative synthesis of the extracted data was employed to analyse, integrate, and synthesise review findings. Study characteristics were firstly tabulated along with extracted outcomes to support the narrative synthesis. A preliminary synthesis was then undertaken by categorising extracted data clusters by reference/country, data source, study objective, inclusion criteria, LBP definition and clinical and economic burden of LBP. Using the tabulated data, relationships within and between included studies were then examined visually.

If necessary, costs were converted to US$ using Salary converter [14]. Most of costs have been converted to US$ using purchasing power party and then inflation adjustments were calculated using the suggested country specific inflation index from the from the World Bank Website [15]. Conversions were performed in February 2022. Further, hospitalization rates were the number of LBP patients hospitalised in a given period of follow-up. The higher rate of hospitalisation could mean substantial clinical burden of the condition.

Patient and public involvement

Patients and the public were not involved in the design or planning of the study.

Results

This systematic review generated 3,074 records in Scopus (n = 51), PubMed (n = 2,036), Embase (n = 700) and Medline, AMED, CINAHL, PsycINFO (n = 287) (Figure 1). Of these, 278 were duplicates. Following screening by titles and abstracts 2,766 studies were excluded, leaving 30 articles for further full text review. No article was added through manual search of the bibliographies of relevant articles. After reading the full text only 9 met the inclusion criteria and were eligible for analysis. The list of the excluded studies was also reported (Appendix 2). The mean NOS score of the included studies was 4, with an average from 3 to 6 (Table 1). The NOS score showed all the included studies do not show comparability of cohorts on the basis of age, sex the design, and analysis.

....................Figure 1 about here.................

Characteristics of the included studies

The characteristics of the included studies are presented in Table 1. The clinical and economic burden of LBP studies were conducted in Nigeria (n = 3), Ethiopia (n = 1), Brazil (n = 2), China (n = 1), Republic of Serbia (n = 1), and Argentina (n = 1). Of the 9 eligible studies included in our review, 3 studies [16, 17, 18] reported the clinical impact of LBP in LMICs. Whereas the remaining studies reported the economic burden of LBP. The data sources of the included studies were interviews, questionnaire, population based cross-sectional study and medical records. Except three studies [16, 19, 20], all the included studies reported LBP Classification or diagnosis.

For peer review only - http://bmjopen.bmj.com/site/about/guidelines.xhtml

Table 1: Characteristics of clinical and economic burden studies included in the review

Reference/ Country	Data source	Study objective	Inclusion criteria	Classification or diagnosis LBP	Conflict of interest	Funding source	NOS/9
Beyera et al., [16]/ Ethiopia	Interview	To analyse hospital admission and associated factors	Individuals with LBP of ≥ 18 years of age. Presented to health care facility for LBP in the past 1-year	Not provided	No	Not stated	3
Carregaro et al., [17]/ Brazil	National databases, considering the period 2012–2016.	To estimate the healthcare expenditure and productivity losses related to LBP	≥ 19 years	ICD-10 codes	No	Yes	6
Laffont et al. [18]/ Argentina	The Statistics and Information Department of the Ministry of Health	To analyse the impact of LBP on hospitalisations between 2006 and 2010.	Either adult LBP or lumbosciatica (≥ 18 years)	ICD-10 codes	No	No	6
Odole et al. [19/ Nigeria	Questionnaire	To determine the economic burden of LBP patients in Ibadan	Individuals with LBP receiving physiotherapy in Ibadan	Not provided	Not stated	Not stated	3
do Socorro et al., [20]/Brazil	Questionnaire	To assess resource utilization in the diagnosis, management, and hospitalization of patients	Participants were rheumatologists attending a national rheumatology medical congress.	Not provided	Not stated	No	4
Bello & Muhammad [21] /Nigeria	Questionnaire	To estimate the cost of LBP management	Patients with LBP, allow them to carry out activities for daily living even under difficulties.	ICD-9 codes	No	No	3
Birabi et al. [22]/ Nigeria	Medical records of Hospitals (220 patients)	To report the results of a retrospective study on the direct cost burden of LBP between 2010-2013	Patients whose care commenced from acute care stages, admitted where necessary.	ICD-10 codes	Not stated	Not stated	5
Lin et al. [23]/ Republic of China	Interview	To estimate the 12- month economic cost of LBP among female (1990 to 1991)	Nurses who had experienced LBP within the 12-month period	ICD-10 codes	No	No	3
Radoičlc et al. [24]/ Republic of Serbia	Interview (April 2016 to April 2017).	To estimate the cost of LBP	Diagnosed as M54, which refers to lumbar pain (Dorsalgia)	ICD-10 codes	No	Partially funded	4

Note: ICD = International Classification of Diseases; NOS = Newcastle–Ottawa quality assessment scale

For peer review only - http://bmjopen.bmj.com/site/about/guidelines.xhtml

Definition of LBP

The definition of LBP adopted by the included studies are presented in Table 2. Except two studies [18, 20], all the included studies provided the definition of LBP. The definition adopted by each included study are not the same. For example, two studies [17, 24] used ICD-codes to define LBP. Other study [16] adopted the frequency of LBP report to health institution.

Table 2: Definition of LBP in the included studies

Study	Definition of LBP
Beyera et al., [16]	Individuals who reported at least 1 presentation to any of the health institutions for their LBP in the past 1-year.
Carregaro et al., [17]	ICD-10 codes were used to define the presence of LBP: M40.4 (Other lordosis); M40.5 (Lordosis, unspecified); M51 (Other intervertebral disc disorders); M54.1 (Radiculopathy); M54.3 (Sciatica); M54.4 (Lumbago with sciatica); M54.5 (Low back pain); M54.8 (Other dorsalgia); and M54.9 (Dorsalgia, unspecified).
Laffont et al. [18]	Not provided
Odole et al. [19]	Patients with mechanical LBP who were receiving physiotherapy on an outpatient
do Socorro et al., [20]	Not provided
Bello & Muhammad [21]	Participants diagnosed and managed for LBP in secondary and tertiary hospitals for at least one-year duration. The pain was low in the back and allow them to carry out activities for daily living even under difficulties.
Birabi et al. [22]	Patients whose ages ranged between 26 and 65years and were managed from acute care period for LBP (without discharge against medical advice) as well as were followed up on out-patient basis without default within the study period.
Lin et al. [23]	The following definition of pain were used: minor: pain occurs when carrying 20 kg of weight for 10 minutes or more; 2) moderate: pain occurs when lifting 20 kg of weight; 3) serious: pain occur; when lifting less than 20 kg of weight; 4) severe: pain occurs when doing any task; 5) maximum: pain occurs even while at rest.
Radoičlc et al. [24]	Patients should have been diagnosed as M54, which refers to lumbar pain (Dorsalgia).

For peer review only - http://bmjopen.bmj.com/site/about/guidelines.xhtml

Hospitalisation

A total of 3 studies reported data on rates of hospitalisation for LBP (Table 3). The rates of hospitalisation ranged between 13.4% to 18.7%. The highest rate of hospitalisation (18.7%) for LBP was reported in Argentina [18].

Table 3: Rate of hospitalisation for LBP patients

Study	Number of participants	Year	Hospitalizations for LBP
Beyera et al. [16]	543 (M = 316; F = 205)	Between June and November 2018	14.4%, [95% CI 11.4-17.3]<br>With an average length of stay 7.4 days, 95% CI 6.4-8.8
Carregaro et al. [17]	886,523 diagnostic imaging procedures for LBP	2012 to 2016	13.4% (inpatient admissions)
Laffont et al. [18]	A total of 154,430 discharges in which patients of any age were hospitalised	2006 and 2010	18.7%,<br>With an average length of stay in the hospital of 3.8 days
do Socorro et al. [20]	207 rheumatologists (M = 62%; F = 38%)	N/A	An average of 574 patients with LBP were seen per year.

Note: N/A = Not available; M = Male; F = Female

Economic burden

The economic burden of the included studies is provided in Table 4. A total of 6 studies reported average annual cost per patient [16, 19, 21, 22, 23, 24]. One of the included studies reported annual cost per population [18]. Patients with the age range up to 85 years were included in the included studies. Due to variation in methodological approaches, the reported cost estimates were inconsistent across the studies. However, up to an average of annual costs of US$2.2 billion and US$1226.25 per population and per patient level, respectively were reported. Up to $1.7 billion indirect costs were also reported mainly due to work absenteeism [18].

For peer review only

Table 4: Summary of studies that reported patient and population -level total costs for LBP

Reference	Age group (years)	Average annual cost per patient	Inflated 2022 $US
Carregaro et al. [17]	≥ 19	Total costs = US$ 2.2 billion¶ Indirect cost = 1.7 billion ¶ (productivity losses)	Total cost = US$2.5 billion¶
Odole et al. [19]	30 to 80	Total cost = US$1125.27 ± 623.39 Direct costs = US$927.20 ± 600.26. Indirect cost = US$198.08 ± 136.15	Total cost = US$5047.5 ± 2796.3 Direct costs = US$4158.1 ± 2691); Indirect cost = US$888 ± 610.
do Socorro et al. [20]	N/A	30% of patients with LBP would be absent from their work due to the condition.	N/A
Bello & Muhammad, [21]	30 to 70	Total = US$763.27 ± 543.97; Direct cost = US$647.28 ± 438.99; Indirect cost = US$115.99 ± 104.99	Total = US$ 3029.9 ± 2159.1 Direct cost = US$2569.5 ± 1742.6 Indirect cost = 460.1 ± 416.5
Birabi et al. [22]	26 and 65	Total cost = US$1226.25 (government hospitals); US$4884.37 (private hospitals)	#US$3192.53 (government hospitals) #US$12716.41(private hospitals)
Lin et al. [23]	Mean age = 32.2 ± 9.2	Total costs = US$994.4 to US$1406.4.	N/A
Radoičlc et al. [24]	Median, 53.59 (28 – 85)	Total costs = €200.40 ± €86.65, Direct costs = €9.39 ± €6.66. Indirect costs = €182.00 ± €78.66	Total cost = €432.86 ± €187.2 Total indirect costs = €393.12 ± €169.99

Note: ¶ = per population; N/A = Not available

Discussion

To our knowledge this is the first systematic review to analyse the clinical and economic impact of LBP in LMICs. A total of 9 studies from six LMICs were included. Our review indicates that the clinical and economic burden of LBP is substantial in LMICs. The rate of hospitalisation ranged between 13.4% and 18.7% of patients with LBP. The findings of the review also suggest that LBP is associated with an annual cost of US$2.2 billion per population in LMICs. A clearer understanding of the clinical and economic burden of LBP in LMICs is therefore important for policy makers that they may consider appropriate prevention and management strategies for LBP.

Rate of hospitalisation of female patients with LBP was higher (53.7%) than their male counterpart [20]. The reason for this could be epidemiological where the proportion of LBP in female is in a higher proportion than males [25]. The average length of stay in hospital due to LBP in Argentina and Ethiopia was 3.8 days [20] and 7.4 days [17], respectively. The length of stay in hospital is directly associated with high financial impact of LBP, this is because of its healthcare and social costs [26].

We collected evidence of both direct and indirect economic burden of LBP. The overall burden varied based on characteristics of patient populations, geographic region and the methodologies adopted by the studies, this includes the individual patient or the population level perspective. For example, the societal annual costs amounted to US$ 2.2 billion per population where the productivity losses represented 79% of the costs reported in Brazil [18].

For peer review only - http://bmjopen.bmj.com/site/about/guidelines.xhtml

Overall, the considerable clinical and economic burden of LBP in LMICs could be due to its poorly equipped health and social system [27].

There are certain strength and limitations to this study. The main strength of this review is the comprehensiveness of the search terms, screening of numerous data bases, and assessment of methodological quality of the studies. Only English language studies were included. Therefore, it is possible that relevant literature published in other languages may have been excluded. Also, grey literature were not included in the current review, as a result, we cannot be certain that the findings of this review are representative of all the studies undertaken, negative results are less likely to be published which may result in publication bias. The quality of the included studies was evaluated using NOS as moderate, however, the instrument used has some limitations as important methodological issues pertaining to health economics might not have been fully assessed by scale. It was not possible to undertake a meta-analysis for most of the included studies due to the adoption of different time horizons, different clinical outcomes and lack of data regarding mean and SD for costs [28]. Further, the quality of the included studies indicates that there is a need to interpret the clinical and economic burden results with caution.

In conclusion: We found that LBP is associated with significantly high rate of hospitalisation with a clear impact on length of stay in hospital and treatment costs. The findings of our analysis can be used by clinicians, and policymakers to better allocate resources for prevention and management strategies for LBP improve health outcomes and reduce the substantial burden associated with the condition. We also hope our results will be of use to researchers planning to evaluate the cost-effectiveness of various strategies for preventing LBP in LMICs.

Authors contribution: T.G. & F.F. performed electronic searches and assessed title and abstract. C.EM. and U.U. were consulted in case of disagreement. F.F. & T.G. performed the methodological quality assessment and extracted the data. T.G. & C.EM. wrote the original draft preparation. F.F. & U.U. reviewed, revised, and edited this paper. All authors read and approved the final draft of the manuscript**.**

Ethical approval: For this study ethical approval is not required.

Competing interest: The authors have no competing interests to declare.

Funding information: This research did not receive any specific grant from funding agencies in the public, commercial, or not-for-profit sectors.

Informed consent: The participants' written consent was not required in this study because this was a systematic review study.

Data sharing: All data related to this work are available.

For peer review only - http://bmjopen.bmj.com/site/about/guidelines.xhtml

References

1. Urits I, Burshtein A, Sharma M, Testa L, Gold PA, Orhurhu V, Viswanath O, Jones MR, Sidransky MA, Spektor B, Kaye AD. Low back pain, a comprehensive review: pathophysiology, diagnosis, and treatment. Current pain and headache reports. 2019 Mar;23(3):1-0.
2. Tay M, Sian SC, Eow CZ, Ho KL, Ong JH, Sirisena D. Ultrasound-Guided Lumbar Spine Injection for Axial and Radicular Pain: A Single Institution Early Experience. Asian Spine Journal. 2021 Apr;15(2):216.
3. Hoy D, Brooks P, Blyth F, Buchbinder R. The epidemiology of low back pain. Best practice & research Clinical rheumatology. 2010 Dec 1;24(6):769-81.
4. Dagenais S, Caro J, Haldeman S. A systematic review of low back pain cost of illness studies in the United States and internationally. The spine journal. 2008 Jan 1;8(1):8-20.
5. Kuritzky L, Samraj GP. Nonsteroidal anti-inflammatory drugs in the treatment of low back pain. Journal of Pain Research. 2012;5:579.
6. Chou R, Qaseem A, Snow V, Casey D, Cross Jr JT, Shekelle P, Owens DK, Clinical Efficacy Assessment Subcommittee of the American College of Physicians and the American College of Physicians/American Pain Society Low Back Pain Guidelines Panel*. Diagnosis and treatment of low back pain: a joint clinical practice guideline from the American College of Physicians and the American Pain Society. Annals of internal medicine. 2007 Oct 2;147(7):478-91.
7. Koes BW, Van Tulder M, Lin CW, Macedo LG, McAuley J, Maher C. An updated overview of clinical guidelines for the management of non-specific low back pain in primary care. European Spine Journal. 2010 Dec;19(12):2075-94.
8. Vos T, Flaxman AD, Naghavi M, Lozano R, Michaud C, Ezzati M, Shibuya K, Salomon JA, Abdalla S, Aboyans V, Abraham J. Years lived with disability (YLDs) for 1160 sequelae of 289 diseases and injuries 1990–2010: a systematic analysis for the Global Burden of Disease Study 2010. The lancet. 2012 Dec 15;380(9859):2163-96.
9. Hoy D, March L, Brooks P, Blyth F, Woolf A, Bain C, Williams G, Smith E, Vos T, Barendregt J, Murray C. The global burden of low back pain: estimates from the Global Burden of Disease 2010 study. Annals of the rheumatic diseases. 2014 Jun 1;73(6):968-74.
10. Louw QA, Morris LD, Grimmer-Somers K. The prevalence of low back pain in Africa: a systematic review. BMC Musculoskeletal disorders. 2007 Dec;8(1):1-4.
11. Stewart Williams J, Ng N, Peltzer K, Yawson A, Biritwum R, Maximova T, Wu F, Arokiasamy P, Kowal P, Chatterji S. Risk factors and disability associated with low back pain in older adults in low-and middle-

For peer review only - http://bmjopen.bmj.com/site/about/guidelines.xhtml

income countries. Results from the WHO Study on Global AGEing and Adult Health (SAGE). PloS one. 2015 Jun 4;10(6):e0127880.

12. Stang A. Critical evaluation of the Newcastle-Ottawa scale for the assessment of the quality of nonrandomized studies in meta-analyses. European journal of epidemiology. 2010 Sep;25(9):603-5.
13. Suissa S, Ernst P, Kezouh A. Regular use of inhaled corticosteroids and the long term prevention of hospitalisation for asthma. Thorax. 2002 Oct 1;57(10):880-4.
14. Salary converter. Purchasing power parities. 2017 http:// salaryconverter. nigelb. me/ (accessed February 2022).
15. World Bank. World Bank open data. 2022. Available at: https://data.worldbank.org/indicator/FP.CPI. TOTL. Accessed February 2022.
16. Beyera GK, O'Brien J, Campbell S. Hospital admission and associated factors among individuals presenting to healthcare facilities for low back pain in Ethiopia. International Journal of Rheumatic Diseases. 2020 Jun;23(6):763-71.
17. Carregaro RL, Tottoli CR, Rodrigues DD, Bosmans JE, da Silva EN, van Tulder M. Low back pain should be considered a health and research priority in Brazil: Lost productivity and healthcare costs between 2012 to 2016. PloS one. 2020 Apr 1;15(4):e0230902.
18. Laffont M, Sequeira G, Kerzberg EM, Marconi E, Guevel C, de las Mercedes Fernández M. The non-silent epidemic: low back pain as a primary cause of hospitalisation. Rheumatology international. 2016 May;36(5):673-7.
19. Odole AC, Akinpelu AA, Adekanla BA, Obisanya OB. Economic burden of low back pain on patients seen at the outpatient Physiotherapy clinics of secondary and tertiary health institutions in Ibadan. Journal of the Nigeria Society of Physiotherapy. 2011 Dec 28;18(1):43-8.
20. do Socorro Margarido M, Kowalski SC, Natour J, Ferraz MB. Acute low back pain: diagnostic and therapeutic practices reported by Brazilian rheumatologists. Spine. 2005 Mar 1;30(5):567-71.
21. Bello B, Muhammad IT. Estimation of economic cost of low back pain management in Kano, Nigeria. Adv Life Sci Med. 2017;3:01-8.
22. Birabi, BN, Oke, KI, Dienye, PO, & Okafor, UAC. Cost Burden of Low Back Pain Amon Adults in Southern Nigeria. International Journal of Medical and Applied Science, 2014 June 4; 3 (3): 24-29.
23. Lin MR, Tsauo JY, Wang JD. Determinants of economic cost related to low back pain among nurses at a University Hospital. International Journal of Occupational and Environmental Health. 1996 Oct 1;2(4):257-63.
24. Radoičlc M, Božovic B, Ilić K, Jankovic S, AnĐelković J, Kostic M. Pharmacoeconomic aspects of low back pain treatment: Cost of illness study in the republic of Serbia. Acta Medica Portuguesa. 2019 Apr 1.
25. Hoy D, Bain C, Williams G, March L, Brooks P, Blyth F, Woolf A, Vos T, Buchbinder R. A systematic review of the global prevalence of low back pain. Arthritis & Rheumatism. 2012 Jun;64(6):2028-37.
26. Anema JR, Schellart AJ, Cassidy JD, Loisel P, Veerman TJ, Van der Beek AJ. Can cross country differences in return-to-work after chronic occupational back pain be explained? An exploratory analysis

For peer review only - http://bmjopen.bmj.com/site/about/guidelines.xhtml

on disability policies in a six country cohort study. Journal of occupational rehabilitation. 2009 Dec;19(4):419-26.

27. Hartvigsen J, Hancock MJ, Kongsted A, Louw Q, Ferreira ML, Genevay S, Hoy D, Karppinen J, Pransky G, Sieper J, Smeets RJ. What low back pain is and why we need to pay attention. The Lancet. 2018 Jun 9;391(10137):2356-67.

28. Higgins MD, Green RJ, Leeson MS. Optical wireless for intravehicle communications: A channel viability analysis. IEEE Transactions on Vehicular Technology. 2011 Nov 18;61(1):123-9.

For peer review only

Figure 1. Flow diagram of publications included and excluded in the review

For peer review only

For peer review only - http://bmjopen.bmj.com/site/about/guidelines.xhtml

Figure

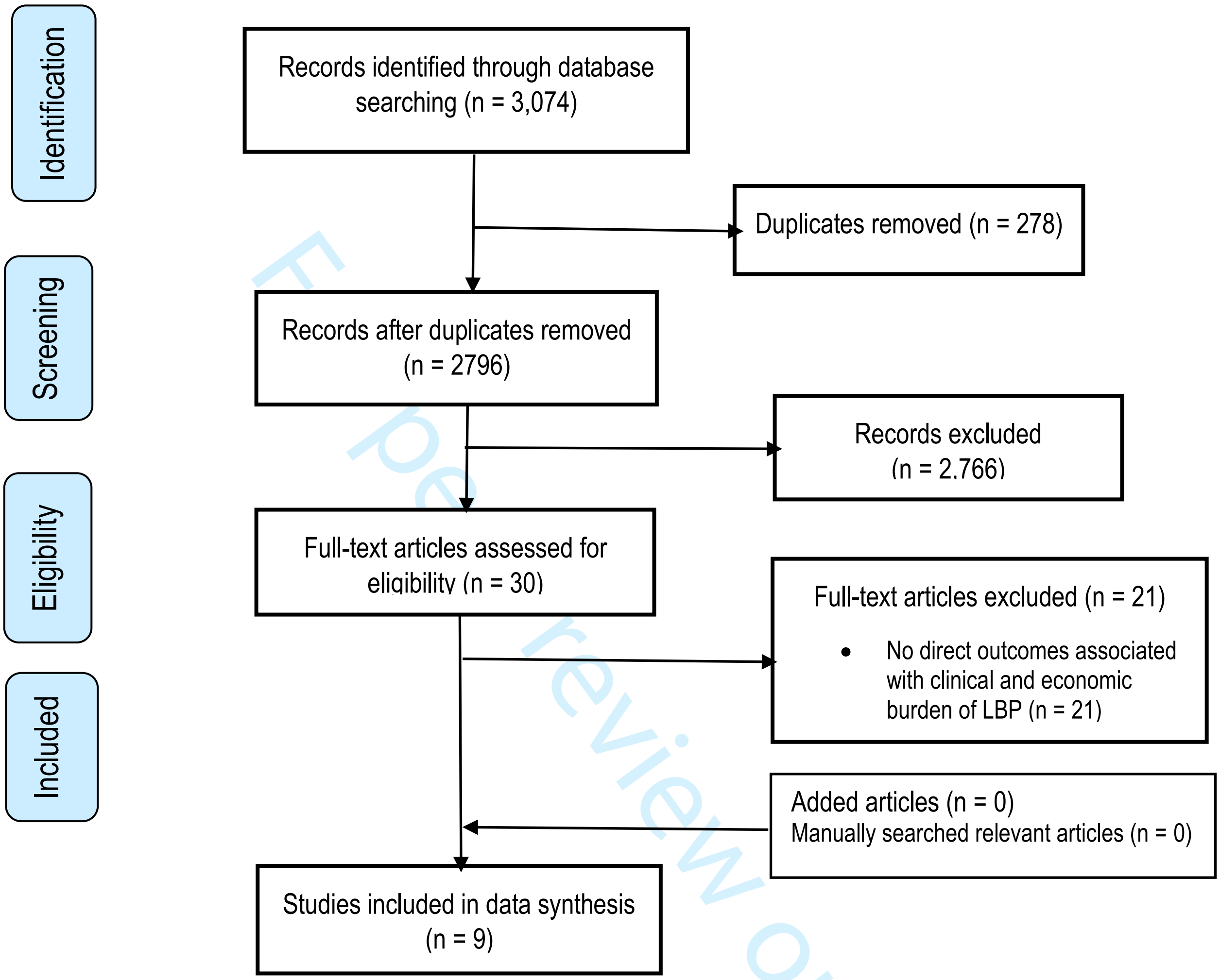

Figure 1. Flow diagram of publications included and excluded in the review

For peer review only - http://bmjopen.bmj.com/site/about/guidelines.xhtml

Appendix 1: Clinical and economic burden search strategy

Appendix 1a: Medline, CINAHL, Embase and AMED (inception to 10th December/2021

Search ID	Search Terms
S23	S21 AND S22
S22	S1 OR S2 OR S3 OR S4 OR S5 OR S6 OR S7 OR S8 OR S9 OR S10 OR S11 OR S12 OR S13 OR S14 OR S15 OR S16 OR S17 OR S18 OR S19 OR S20
S21	AB low back pain
S20	AB hospitalization
S19	AB cost of illness
S18	AB absenteeism
S17	AB ambulatory care
S16	AB drug cost
S15	AB emergency medical services
S14	AB healthcare services
S13	AB nursing services
S12	AB economics
S11	AB physician
S10	AB burden
S9	AB clinical impact
S8	AB utilization
S7	AB burden of illness
S6	AB cost
S5	AB nursing costs
S4	AB physician cost
S3	AB physician visits
S2	AB emergency department visits
S1	AB years lived with disability

For peer review only

Appendix 1b: Scopus (inception to 10th December/2021

Search ID	Search Terms
1	TITLE-ABS-KEY (low AND back AND pain)
2	TITLE-ABS-KEY (hospitalisation)
3	TITLE-ABS-KEY (cost AND of AND illness)
4	TITLE-ABS-KEY (absenteeism)
5	TITLE-ABS-KEY (ambulatory AND care)
6	TITLE-ABS-KEY (drug AND costs)
7	TITLE-ABS-KEY (emergency AND medical AND services)
8	TITLE-ABS-KEY (healthcare AND costs)
9	TITLE-ABS-KEY (nursing AND services)
10	TITLE-ABS-KEY (economics)
11	TITLE-ABS-KEY (physicians)
12	TITLE-ABS-KEY (burden)
13	TITLE-ABS-KEY (clinical AND impact)
14	TITLE-ABS-KEY (utilization)
15	TITLE-ABS-KEY (burden AND of AND illness)
16	TITLE-ABS-KEY (cost)
17	TITLE-ABS-KEY (nursing AND cost)
18	TITLE-ABS-KEY (physician AND cost)
19	TITLE-ABS-KEY (physician AND visit)
20	(TITLE-ABS-KEY (hospitalisation)) OR (TITLE-ABS-KEY (cost AND of AND illness)) OR (TITLE-ABS-KEY (absenteeism)) OR (TITLE-ABS-KEY (ambulatory AND care)) OR (TITLE-ABS-KEY (drug AND costs)) OR (TITLE-ABS-KEY (emergency AND medical AND services)) OR (TITLE-ABS-KEY (healthcare AND costs)) OR (TITLE-ABS-KEY (nursing AND services)) OR (TITLE-ABS-KEY (economics)) OR (TITLE-ABS-KEY (physicians)) OR (TITLE-ABS-KEY (burden)) OR (TITLE-ABS-KEY (clinical AND impact)) OR (TITLE-ABS-KEY (utilization)) OR (TITLE-ABS-KEY (burden AND of AND illness)) OR (TITLE-ABS-KEY (cost)) OR (TITLE-ABS-KEY (nursing AND cost)) OR (TITLE-ABS-KEY (physician AND cost)) OR (TITLE-ABS-KEY (physician AND visit))
21	(TITLE-ABS-KEY (low AND back AND pain)) AND ((TITLE-ABS-KEY (hospitalisation)) OR (TITLE-ABS-KEY (cost AND of AND illness)) OR (TITLE-ABS-KEY (absenteeism)) OR (TITLE-ABS-KEY (ambulatory AND care)) OR (TITLE-ABS-KEY (drug AND costs)) OR (TITLE-ABS-KEY (emergency AND medical AND services)) OR (TITLE-ABS-KEY (healthcare AND costs)) OR (TITLE-ABS-KEY (nursing AND services)) OR (TITLE-ABS-KEY (economics)) OR (TITLE-ABS-KEY (physicians)) OR (TITLE-ABS-KEY (burden)) OR (TITLE-ABS-KEY (clinical AND impact)) OR (TITLE-ABS-KEY (utilization)) OR (TITLE-ABS-KEY (burden AND of AND illness)) OR (TITLE-ABS-KEY (cost)) OR (TITLE-ABS-KEY (nursing AND cost)) OR (TITLE-ABS-KEY (physician AND cost)) OR (TITLE-ABS-KEY (physician AND visit)))

Appendix 1c: PubMed (inception to 10th December/2021

Query	Search Details
((((((((((((((((Hospitalisation[Title/Abstract] AND ((humans[Filter]) AND (english[Filter]))) OR (cost of illness[Title/Abstract] AND ((humans[Filter]) AND (english[Filter])))) OR (Absenteeism[Title/Abstract] AND ((humans[Filter]) AND (english[Filter])))) OR (Ambulatory care[Title/Abstract] AND ((humans[Filter]) AND (english[Filter])))) OR (Drug costs[Title/Abstract] AND ((humans[Filter]) AND (english[Filter])))) OR (Emergency medical services[Title/Abstract] AND ((humans[Filter]) AND (english[Filter])))) OR (healthcare costs[Title/Abstract] AND ((humans[Filter]) AND (english[Filter])))) OR (nursing services[Title/Abstract] AND ((humans[Filter]) AND (english[Filter])))) OR (economics[Title/Abstract] AND ((humans[Filter]) AND (english[Filter])))) OR (physicians[Title/Abstract] AND ((humans[Filter]) AND (english[Filter])))) OR (burden[Title/Abstract] AND ((humans[Filter]) AND (english[Filter])))) OR (clinical impact[Title/Abstract] AND ((humans[Filter]) AND (english[Filter])))) OR (Utilization[Title/Abstract] AND ((humans[Filter]) AND (english[Filter])))) OR (burden of illness[Title/Abstract] AND ((humans[Filter]) AND (english[Filter])))) OR (cost[Title/Abstract] AND ((humans[Filter]) AND (english[Filter])))) OR (nursing cost[Title/Abstract] AND ((humans[Filter]) AND (english[Filter])))) OR (physician visit[Title/Abstract] AND ((humans[Filter]) AND (english[Filter])))) AND (low back pain[Title/Abstract] AND ((humans[Filter]) AND (english[Filter])))	((("Hospitalisation"[Title/Abstract] AND ("humans"[MeSH Terms] AND "english"[Language])) OR ("cost of illness"[Title/Abstract] AND ("humans"[MeSH Terms] AND "english"[Language])) OR ("Absenteeism"[Title/Abstract] AND ("humans"[MeSH Terms] AND "english"[Language])) OR ("ambulatory care"[Title/Abstract] AND ("humans"[MeSH Terms] AND "english"[Language])) OR ("drug costs"[Title/Abstract] AND ("humans"[MeSH Terms] AND "english"[Language])) OR ("emergency medical services"[Title/Abstract] AND ("humans"[MeSH Terms] AND "english"[Language])) OR ("healthcare costs"[Title/Abstract] AND ("humans"[MeSH Terms] AND "english"[Language])) OR ("nursing services"[Title/Abstract] AND ("humans"[MeSH Terms] AND "english"[Language])) OR ("economics"[Title/Abstract] AND ("humans"[MeSH Terms] AND "english"[Language])) OR ("physicians"[Title/Abstract] AND ("humans"[MeSH Terms] AND "english"[Language])) OR ("burden"[Title/Abstract] AND ("humans"[MeSH Terms] AND "english"[Language])) OR ("clinical impact"[Title/Abstract] AND ("humans"[MeSH Terms] AND "english"[Language])) OR ("Utilization"[Title/Abstract] AND ("humans"[MeSH Terms] AND "english"[Language])) OR ("burden of illness"[Title/Abstract] AND ("humans"[MeSH Terms] AND "english"[Language])) OR ("cost"[Title/Abstract] AND ("humans"[MeSH Terms] AND "english"[Language])) OR ("nursing cost"[Title/Abstract] AND ("humans"[MeSH Terms] AND "english"[Language])) OR ("physician visit"[Title/Abstract] AND ("humans"[MeSH Terms] AND "english"[Language]))) AND ("low back pain"[Title/Abstract] AND ("humans"[MeSH Terms] AND "english"[Language]))) AND ((humans[Filter]) AND (english[Filter]))
low back pain[Title/Abstract]	("low back pain"[Title/Abstract]) AND ((humans[Filter]) AND (english[Filter]))
physician visit[Title/Abstract]	("physician visit"[Title/Abstract]) AND ((humans[Filter]) AND (english[Filter]))
nursing cost[Title/Abstract]	("nursing cost"[Title/Abstract]) AND ((humans[Filter]) AND (english[Filter]))
cost[Title/Abstract]	("cost"[Title/Abstract]) AND ((humans[Filter]) AND (english[Filter]))
burden of illness[Title/Abstract]	("burden of illness"[Title/Abstract]) AND ((humans[Filter]) AND (english[Filter]))
utilization[Title/Abstract]	("Utilization"[Title/Abstract]) AND ((humans[Filter]) AND (english[Filter]))
clinical impact[Title/Abstract]	("clinical impact"[Title/Abstract]) AND ((humans[Filter]) AND (english[Filter]))
burden[Title/Abstract]	("burden"[Title/Abstract]) AND ((humans[Filter]) AND (english[Filter]))
physicians[Title/Abstract]	("physicians"[Title/Abstract]) AND ((humans[Filter]) AND (english[Filter]))
economics[Title/Abstract]	("economics"[Title/Abstract]) AND ((humans[Filter]) AND (english[Filter]))
nursing services[Title/Abstract]	("nursing services"[Title/Abstract]) AND ((humans[Filter]) AND (english[Filter]))
healthcare costs[Title/Abstract]	("healthcare costs"[Title/Abstract]) AND ((humans[Filter]) AND (english[Filter]))
emergency medical services[Title/Abstract]	("emergency medical services"[Title/Abstract]) AND ((humans[Filter]) AND (english[Filter]))
Drug costs[Title/Abstract]	("drug costs"[Title/Abstract]) AND ((humans[Filter]) AND (english[Filter]))
Ambulatory care[Title/Abstract]	("ambulatory care"[Title/Abstract]) AND ((humans[Filter]) AND (english[Filter]))
Absenteeism[Title/Abstract]	("Absenteeism"[Title/Abstract]) AND ((humans[Filter]) AND (english[Filter]))
cost of illness[Title/Abstract]	("cost of illness"[Title/Abstract]) AND ((humans[Filter]) AND (english[Filter]))
hospitalisation[Title/Abstract]	("Hospitalisation"[Title/Abstract]) AND ((humans[Filter]) AND (english[Filter]))

Appendix 2: List of excluded studies

Study	Country	Design	Aim	Justification for exclusion
Safiri et al., 2021	Global	Model	To determine the burden of other musculoskeletal disorders between 1990 and 2017 at the global, regional and national level across 195 countries and territories	Estimate not for low back pain
Barreto & Sá, 2019	Brazil	Questionnaire	To estimate indirect cost related to chronic pain from an employer's perspective	Estimate not for low back pain
Du et al., 2019	China	CR	To systematically explore the correlates of emotional distress in patients with chronic low back pain	Not clinical or economic burden study.
Driscoll et al., 2014	Global	Servery	To quantify the burden arising from low back pain (LBP) due to occupational exposure to ergonomic risk factors.	Not clinical or economic burden study.
Hoy et al., 2014	Global	Survey	To estimate the global burden of LBP.	Not related to the research topic
Smith et al., 2014	Global	Systematic reviews	To estimate disability from the remainder of musculoskeletal (MSK) disorders	Not clinical or economic burden study
Vos et al., 2012	Worldwide	Model	To describe the approach to undertaking past burden of disease assessments with the available evidence.	Not related
Moradi-Lakeh et al., 2017	EMR	Systematic analysis	To report the burden of musculoskeletal disorders in the Eastern Mediterranean Region.	Not clinical or economic burden study
de David et al., 2020	Brazil	Model	To describe the current status and trends of the burden due to LBP in Brazil	Prevalence and incidence
Amaefule et al., 2021	Nigeria	CR	To estimate the burden of chronic musculoskeletal Disorders amongst patients attending orthopaedic outpatients' clinic	Ful article not available
Aminde et al., 2020	Cameroon	CR	To evaluate health-related quality of life (HRQoL) and its determinants in chronic low back pain patients in Cameroon.	Not clinical or economic burden study
Dagenais et al., 2008	USA	CR	To conduct a systematic review of LBP cost of illness studies	A systematic review
Wu et al., 2019	China	CR	To quantify the prevalence and years lived with disability (YLDs) caused by LBP in China	Not clinical or economic burden study
Dienye et al., 2016	Nigeria	CR	To determine the prevalence of LBP	Prevalence and incidence
Galukande et al., 2006	Uganda	CR	To assess and document the disability associated with low back pain in terms of sick leave	Not clinical or economic burden study
Faezi et al., 2020	Iran	Interviews	To evaluate health-care access and utilization among patients with LBP in Iran	Not clinical or economic burden study
Igwesi-Chidobe et al., 2019	Nigeria	Questionnaire	To assess self-reported disability in a low-literate population with chronic LBP	Not clinical or economic burden study
Michalik et al., 2015	Poland	CR	To present the epidemiology data on back pain in Poland	Prevalence and incidence
Carregaro et al., 2019	Brazil	Prevalence-based	To estimate the direct healthcare costs of spinal disorders in Brazil over 2016.	Not low back pain
Kisa et al., 2020	Global	Systematic analysis	To provides an overview of the influence of occupational risk factors on the global burden of disease	Not low back pain
Maetzel & Li, 2002	N/A	Systematic review	To gain a better understanding of the societal costs of LBP	A review of studies

EMR = Eastern Mediterranean Region; CR = Cross sectional

PRISMA 2020 Checklist

Section and Topic	Item #	Checklist item	Location where item is reported
**TITLE**			
Title	1	Identify the report as a systematic review.	1
**ABSTRACT**			
Abstract	2	See the PRISMA 2020 for Abstracts checklist.	2
**INTRODUCTION**			
Rationale	3	Describe the rationale for the review in the context of existing knowledge.	3
Objectives	4	Provide an explicit statement of the objective(s) or question(s) the review addresses.	3
**METHODS**			
Eligibility criteria	5	Specify the inclusion and exclusion criteria for the review and how studies were grouped for the syntheses.	4
Information sources	6	Specify all databases, registers, websites, organisations, reference lists and other sources searched or consulted to identify studies. Specify the date when each source was last searched or consulted.	4
Search strategy	7	Present the full search strategies for all databases, registers and websites, including any filters and limits used.	4
Selection process	8	Specify the methods used to decide whether a study met the inclusion criteria of the review, including how many reviewers screened each record and each report retrieved, whether they worked independently, and if applicable, details of automation tools used in the process.	4
Data collection process	9	Specify the methods used to collect data from reports, including how many reviewers collected data from each report, whether they worked independently, any processes for obtaining or confirming data from study investigators, and if applicable, details of automation tools used in the process.	4
Data items	10a	List and define all outcomes for which data were sought. Specify whether all results that were compatible with each outcome domain in each study were sought (e.g. for all measures, time points, analyses), and if not, the methods used to decide which results to collect.	5
	10b	List and define all other variables for which data were sought (e.g. participant and intervention characteristics, funding sources). Describe any assumptions made about any missing or unclear information.	5
Study risk of bias assessment	11	Specify the methods used to assess risk of bias in the included studies, including details of the tool(s) used, how many reviewers assessed each study and whether they worked independently, and if applicable, details of automation tools used in the process.	4
Effect measures	12	Specify for each outcome the effect measure(s) (e.g. risk ratio, mean difference) used in the synthesis or presentation of results.	N/R
Synthesis methods	13a	Describe the processes used to decide which studies were eligible for each synthesis (e.g. tabulating the study intervention characteristics and comparing against the planned groups for each synthesis (item #5)).	5
	13b	Describe any methods required to prepare the data for presentation or synthesis, such as handling of missing summary statistics, or data conversions.	5
	13c	Describe any methods used to tabulate or visually display results of individual studies and syntheses.	5
	13d	Describe any methods used to synthesize results and provide a rationale for the choice(s). If meta-analysis was performed, describe the model(s), method(s) to identify the presence and extent of statistical heterogeneity, and software package(s) used.	N/R
	13e	Describe any methods used to explore possible causes of heterogeneity among study results (e.g. subgroup analysis, meta-regression).	N/R
	13f	Describe any sensitivity analyses conducted to assess robustness of the synthesized results.	N/R
Reporting bias assessment	14	Describe any methods used to assess risk of bias due to missing results in a synthesis (arising from reporting biases).	5
Certainty	15	Describe any methods used to assess certainty (or confidence) in the body of evidence for an outcome.	N/R

For peer review only - http://bmjopen.bmj.com/site/about/guidelines.xhtml

For peer review only

PRISMA 2020 Checklist

Section and Topic	Item #	Checklist item	Location where item is reported
assessment			
**RESULTS**			
Study selection	16a	Describe the results of the search and selection process, from the number of records identified in the search to the number of studies included in the review, ideally using a flow diagram.	5
	16b	Cite studies that might appear to meet the inclusion criteria, but which were excluded, and explain why they were excluded.	5
Study characteristics	17	Cite each included study and present its characteristics.	6
Risk of bias in studies	18	Present assessments of risk of bias for each included study.	6
Results of individual studies	19	For all outcomes, present, for each study: (a) summary statistics for each group (where appropriate) and (b) an effect estimate and its precision (e.g. confidence/credible interval), ideally using structured tables or plots.	6
Results of syntheses	20a	For each synthesis, briefly summarise the characteristics and risk of bias among contributing studies.	6
	20b	Present results of all statistical syntheses conducted. If meta-analysis was done, present for each the summary estimate and its precision (e.g. confidence/credible interval) and measures of statistical heterogeneity. If comparing groups, describe the direction of the effect.	N/R
	20c	Present results of all investigations of possible causes of heterogeneity among study results.	N/R
	20d	Present results of all sensitivity analyses conducted to assess the robustness of the synthesized results.	N/R
Reporting biases	21	Present assessments of risk of bias due to missing results (arising from reporting biases) for each synthesis assessed.	N/R
Certainty of evidence	22	Present assessments of certainty (or confidence) in the body of evidence for each outcome assessed.	N/R
**DISCUSSION**			
Discussion	23a	Provide a general interpretation of the results in the context of other evidence.	8
	23b	Discuss any limitations of the evidence included in the review.	8
	23c	Discuss any limitations of the review processes used.	8
	23d	Discuss implications of the results for practice, policy, and future research.	8
**OTHER INFORMATION**			
Registration and protocol	24a	Provide registration information for the review, including register name and registration number, or state that the review was not registered.	3
	24b	Indicate where the review protocol can be accessed, or state that a protocol was not prepared.	3
	24c	Describe and explain any amendments to information provided at registration or in the protocol.	N/R
Support	25	Describe sources of financial or non-financial support for the review, and the role of the funders or sponsors in the review.	9
Competing interests	26	Declare any competing interests of review authors.	9
Availability of data, code and other materials	27	Report which of the following are publicly available and where they can be found: template data collection forms; data extracted from included studies; data used for all analyses; analytic code; any other materials used in the review.	N/R

For peer review only - http://bmjopen.bmj.com/site/about/guidelines.xhtml

From: Page MJ, McKenzie JE, Bossuyt PM, Boutron I, Hoffmann TC, Mulrow CD, et al. The PRISMA 2020 statement: an updated guideline for reporting systematic reviews. BMJ 2021;372:n71. doi: 10.1136/bmj.n71

For peer review only

PRISMA 2020 Checklist

http://www.prisma-statement.org/

For peer review only
